# A rapidly evolving secretome builds and patterns a sea shell

**DOI:** 10.1186/1741-7007-4-40

**Published:** 2006-11-22

**Authors:** Daniel J Jackson, Carmel McDougall, Kathryn Green, Fiona Simpson, Gert Wörheide, Bernard M Degnan

**Affiliations:** 1 School of Integrative Biology, University of Queensland, Brisbane Qld 4072, Australia; 2 Department of Geobiology, Geoscience Centre, University of Göttingen, Goldschmidtstr.3, 37077 Göttingen, Germany; 3 Institute of Molecular Biosciences, University of Queensland, Brisbane Qld 4072, Australia; 4 Department of Zoology, University of Oxford, Tinbergen Bldg., South Parks Road, Oxford OX1 3PS, UK

## Abstract

**Background:**

Instructions to fabricate mineralized structures with distinct nanoscale architectures, such as seashells and coral and vertebrate skeletons, are encoded in the genomes of a wide variety of animals. In mollusks, the mantle is responsible for the extracellular production of the shell, directing the ordered biomineralization of CaCO_3 _and the deposition of architectural and color patterns. The evolutionary origins of the ability to synthesize calcified structures across various metazoan taxa remain obscure, with only a small number of protein families identified from molluskan shells. The recent sequencing of a wide range of metazoan genomes coupled with the analysis of gene expression in non-model animals has allowed us to investigate the evolution and process of biomineralization in gastropod mollusks.

**Results:**

Here we show that over 25% of the genes expressed in the mantle of the vetigastropod *Haliotis asinina *encode secreted proteins, indicating that hundreds of proteins are likely to be contributing to shell fabrication and patterning. Almost 85% of the secretome encodes novel proteins; remarkably, only 19% of these have identifiable homologues in the full genome of the patellogastropod *Lottia scutum*. The spatial expression profiles of mantle genes that belong to the secretome is restricted to discrete mantle zones, with each zone responsible for the fabrication of one of the structural layers of the shell. Patterned expression of a subset of genes along the length of the mantle is indicative of roles in shell ornamentation. For example, *Has-sometsuke *maps precisely to pigmentation patterns in the shell, providing the first case of a gene product to be involved in molluskan shell pigmentation. We also describe the expression of two novel genes involved in nacre (mother of pearl) deposition.

**Conclusion:**

The unexpected complexity and evolvability of this secretome and the modular design of the molluskan mantle enables diversification of shell strength and design, and as such must contribute to the variety of adaptive architectures and colors found in mollusk shells. The composition of this novel mantle-specific secretome suggests that there are significant molecular differences in the ways in which gastropods synthesize their shells.

## Background

The ability to synthesize rigid, mineralized structures is an essential trait to the majority of metazoan taxa. Vertebrates, echinoderms, mollusks, arthropods, brachiopods, bryozoans, annelids, cnidarians and sponges, amongst others, construct a spectacular diversity of endo- and exo-skeletons as well as sensory and protective structures from a range of minerals [1]. The importance of this trait is highlighted by the observation that the so called 'Cambrian explosion' was accompanied by the diversification of biomineralization mechanisms [2-4], despite the fact that several lineages possessed this ability before the end of the Proterozoic [5]. It is currently unknown whether the molecular mechanisms used to create these structures have been inherited from an ancestral biomineralization repertoire, invented de novo, or are the result of an unprecedented lateral genetic transfer [6].

The evolutionary origins, mode of construction, patterning and physical properties of the molluskan shell have held the attention of scientists for centuries, however the molecular mechanisms by which these structures are constructed are only now beginning to be elucidated [7-9]. The mollusk shell is assembled extracellularly and is an ensemble of CaCO_3_ and organic macromolecules (proteins, glycoproteins, lipids and polysaccharides), which are secreted by the mantle epithelium. The anterior edge of the mantle tissue underlies the lip of the shell and directs the ordered biomineralization of the different structural layers of the shell and controls the patterning of architectural and color features. While the structure and function of a number of shell matrix proteins have recently been characterized [7,10-17], the regulatory mechanisms that govern these shell-building processes remain largely unknown.

It has long been acknowledged that the diversity of shell types found in gastropod, bivalve and scaphopod mollusks are achieved through the ordered secretion of proteins and other molecules along the length of mantle [18-21], however the full complexity and role of differential gene activity in the mantle remains undescribed. The color, structure and geometric pattern of a sea shell is a historical record of the incorporation of proteins into the shell matrix and onto its surface, and directly reflects the gene expression activity of the mantle during the life of a mollusk [22]. Using the vetigastropod *Haliotis asinina *(tropical abalone) as a model, we sought to determine the complexity of the mantle transcriptome. Abalone shells are composed of three structurally-distinct layers: (i) the inner nacreous (flat pearl) layer, consisting of layers of aragonitic tablets encased within organic sheaths; (ii) the calcitic prismatic layer, also containing organic macromolecules; and (iii) the outer periostracum, a thin organic veneer that protects and decorates the shell [23]. The anterior edge of the abalone mantle epithelium is convoluted and partitioned into discrete zones that produce each of these layers [24]. Within each of these zones are a number of cell types, which contribute to the construction and patterning of the shell [25,26].

Here we assess the complexity of gene expression in the *H. asinina *mantle, and explore the regulatory and structural factors that contribute to the construction of the shell. Previous studies have demonstrated that the organic component of the shell (often comprising less than 5% by dry weight) is essential to its construction, and confers its remarkable physical properties. For example, Lustrin-A [11,27] is thought to impart fracture resistant, elastomeric properties to the nacreous layer, while macromolecules isolated from calcitic or nacreous environments can direct the type of polymorph of CaCO_3 _that will be deposited *in vitro *[28-30]. Unfortunately, shell matrix proteins are often insoluble, highly acidic or complexed with minerals, making their purification very difficult [31]. To gain a broader understanding of the molecular processes that underlie seashell construction, we have analyzed expressed sequence tags (ESTs) from the mantle of juvenile *H. asinina*. This approach allows for the identification of gene products that are not necessarily incorporated into the shell, but are nonetheless crucial for CaCO_3 _precipitation and other biomineralization events within the pallial space adjacent to the mantle. Other mantle-localized, secreted gene products not involved in biomineralization will also be detected by this methodology. We have compared this EST set with the recently sequenced *L. scutum *(Patellogastropoda) genome in order to infer the degree of evolutionary conservation between shell building secretomes within one molluskan class.

## Results and discussion

### Structure of *H. asinina *shell and mantle

**Figure 1:**
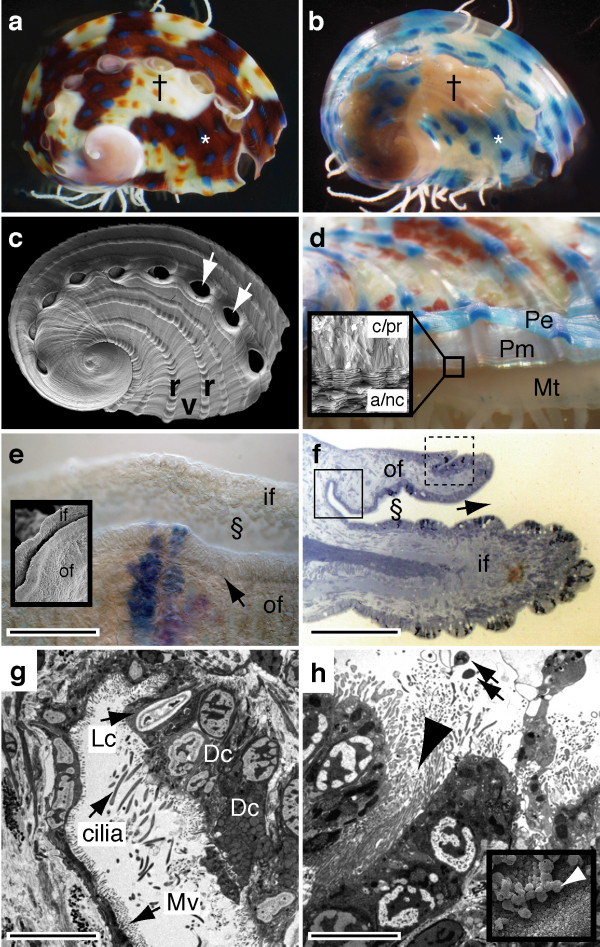
**The tropical abalone as a model for understanding molluskan biomineralization**. (a) Juvenile shell pigmentation follows a simple set of rules; dots are blue when in a red field (*) and orange when in a cream field (†). (b) The decalcified shell reveals a blue color within previous red fields (*). (c) SEM reveals the topographic shell pattern with a ridge (r) and valley (v) organization, and regularly spaced respiratory pores (white arrows). Shells in (a-c) are 5 mm. (d) Pigmentation is restricted to the outer periostracum (Pe), which overlies the protein matrix (Pm). The mantle (Mt) continuously secretes these structures. Insert, SEM cross section of the shell, revealing the calcitic prismatic layer (c/pr) and the inner aragonitic nacreous layer (a/nc). (e) The leading edge of the mantle (dorsal view, anterior top) consists of two folds. Between the ventral most inner (if) and outer folds (of) lies the periostracal groove (§). At the anterior edge of the outer fold there is a crease in the epithelium (arrow). SEM insert of the same region. (f) Section through the mantle edge (dorsal top, anterior right) highlighting the periostracal groove (§). (g) TEM of solid boxed section in (f) reveals light (Lc) and dark (Dc) staining secretory cell types, with microvilli (Mv). (h) TEM section of dashed box in (f) reveals the brush border-like lining of this crease (large arrowhead). Secreted extracellular material (arrows) is evident. Inset shows this extracellular material (white arrowhead) possibly responsible for the initiation of calcification. Scale bars: (e, f) 50 μm; (g, h) 5 μm.

The juvenile shell of *H. asinina *is an ideal model system with which to study the molecular events of shell construction and patterning. A complex but regular chromatic pattern adorns the outermost layer of the shell, the periostracum. Here, a series of dots (either blue or orange) are laid down on top of the ridges of the shell. The color of the dot will be blue if it overlies a brown/red background, or orange if it overlies a cream background (Figure 1a–c). This model system is such that it allows for the spatial mapping of gene expression profiles within the mantle to patterning and structural events at the leading edge of the shell.

The mineralogical composition of the shell is partitioned dorso-ventrally into two major layers; a dorsal calcitic layer and a ventral aragonitic layer (Figure 1d). The mantle epithelium, which secretes the proteins responsible for the construction of these structures, is convoluted at its anterior edge. Between the two main folds (the inner and outer folds) lies the periostracal groove (Figure 1e–g), into which the periostracum is secreted and then extruded onto the dorsal surface of the shell. We have identified a second minor fold within the mantle of *H. asinina *that we have termed the anterior crease of the outer fold (Figure 1e, f and 1h). Although we cannot yet assign a specific function to this structure, the fact that it possesses an abundance of microvilli (Figure 1h) suggests that it is actively secreting substances responsible for shell or periostracum construction.

### Mantle expressed sequence tags

**Figure 2:**
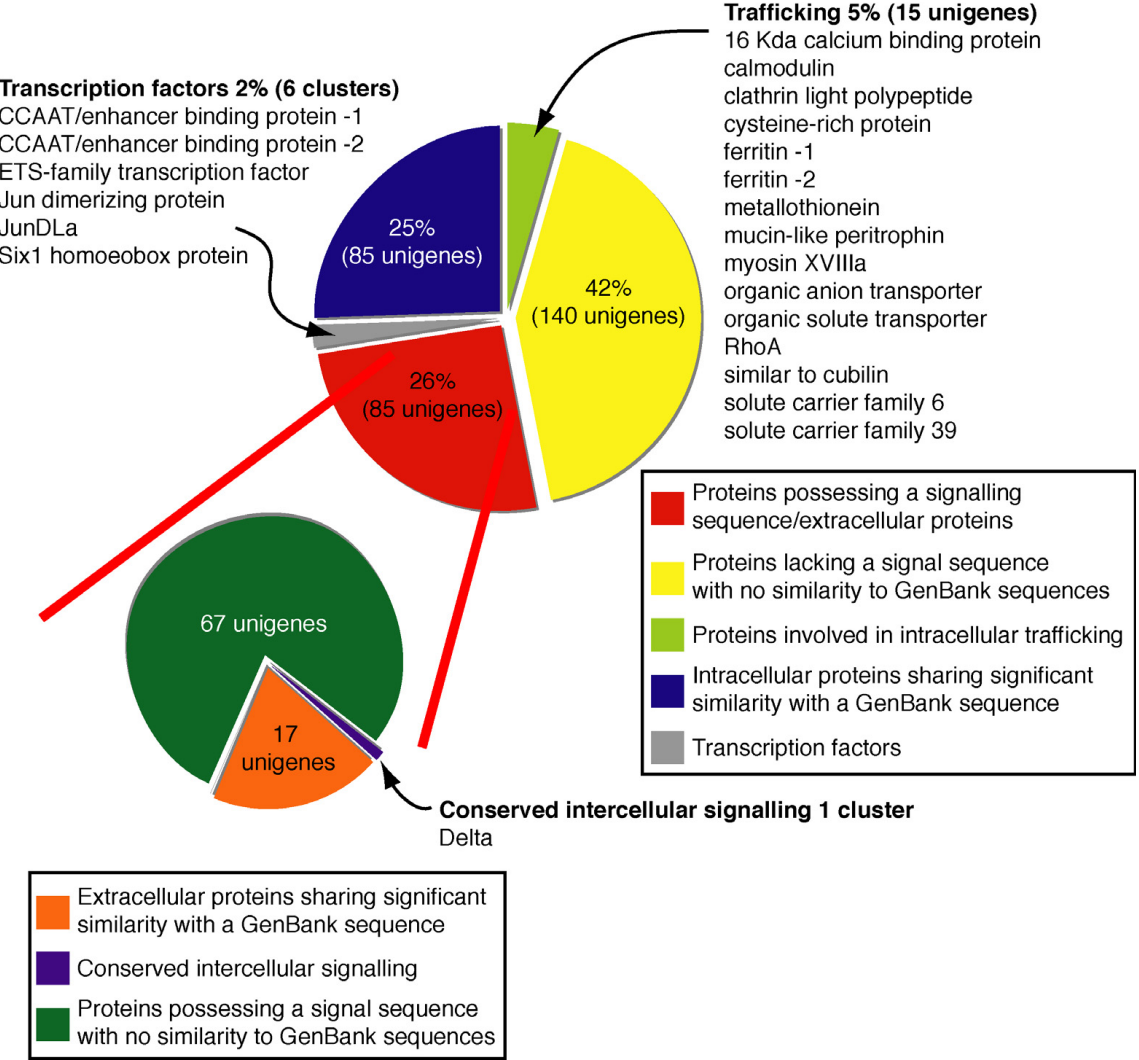
**Categorization of 331 genes expressed in the *H. asinina *mantle**. A total of 331 non-redundant ESTs were clustered using ClustalW and placed into one of five categories according to (i) the presence/absence of a signal sequence, whether they are (ii) a trafficking protein, (iii) a transcription factor, (iv) a signaling molecule or (v) have no identity with existing sequences in GenBank. Proteins predicted to be secreted are further subdivided.

We have sequenced 530 randomly selected clones from a cDNA library constructed from the juvenile mantle tissue of the tropical abalone, *Haliotis asinina *(Linnaeus). These sequences are available under GenBank accession numbers DW986183 to DQ298397. This tissue was taken from juveniles maintained in warm water (26–28°C) that were rapidly depositing shell material, approximately 50 μm per day [32]. From this survey, we have identified 331 unique EST clusters (unigenes) that are expressed in the mantle (Figure 2; Additional file 1). Based on the presence of conserved signal sequences and similarity to secreted proteins in GenBank (Figure 2) we conservatively estimate that 26% of these (85 unigenes) encode secreted proteins. Amongst the 140 unigenes that lack both significant similarity to sequences lodged in public databases (Figure 2) and a signal sequence, some may be secreted via mechanisms alternative to the classical signal peptide pathway. Of the 106 intracellular unigenes that encode proteins with significant similarity to GenBank sequences, 15 encode trafficking and mineral binding proteins, and are likely to represent mechanisms essential for the supply of shell building components. For example, based upon *in situ *hybridization analyses (see below) and previous studies on bivalves [33,34], ferritin and calmodulin are likely to be playing fundamental and evolutionarily ancient roles in shell construction within the Mollusca.

When compared with EST surveys in other metazoan tissues (e.g. various mouse [35], pufferfish [36] and human [36,37] proteomes), a markedly higher proportion (~twofold) of the genes expressed in the abalone mantle encode secreted proteins. By extrapolation, we estimate that hundreds of proteins are released extracellularly from the mantle, and contribute to the fabrication of the shell. This estimate however needs to take into consideration non-biomineralizing secreted proteins that do not possess similarity with previously described GenBank sequences. This estimate is in marked contrast to the current number of biomineralizing proteins isolated from the shells of other gastropods and bivalves. With efforts chiefly focused on the characterization of proteins from the nacreous layer [38-41], fewer than 20 protein families have been shown to contribute to shell formation to date [7,8]. Strikingly, of the 85 unigenes that encode putative secreted proteins in the *Haliotis asinina *mantle, 67 (80% of the secretome) do not share significant similarity to any sequences in GenBank (Figure 2). However, several novel secreted open reading frames (ORFs) possess motifs comparable to proteins in other organisms, such as GGYGLGL repeats – similar to the elasticity regions of Spidroin, an elastomeric spider silk protein [42] – and proline-rich repeats (LXPLSXIPVXXPXAX) as found in plant cell walls [43]. In comparison, 140 of the 246 intracellular genes (57%) do not match sequences in GenBank.

Interestingly, BLAST alignments between *H. asinina *mantle ESTs and genomic trace sequences from the gastropod *L. scutum*, which is estimated to be sequenced to 8× coverage and is currently being assembled [44], reveals that only 13 of the 67 (19%) novel secreted proteins in *Haliotis *appear to have identifiable homologues in the *Lottia *genome (Additional file 2). These 13 genes may be involved in conserved aspects of shell construction in gastropods and possibly other mollusks. In contrast, the remaining 54 novel secreted proteins are likely to either represent genes that evolved after these gastropod lineages split, have been lost in the *Lottia *lineage or a combination of these two scenarios. Likewise, many previously discovered molluskan shell matrix genes do not have clear homologues in the *Lottia *genome (See Additional File 3 for results of these searches against *Lottia *genome traces). The few shell matrix proteins, such as Lustrin [11], Perlucin [45] and Mucoperlin [46], are likely to be conserved components of molluskan shells, although their general function in shell fabrication and evolutionary origins are currently unknown. Together, these data suggest that the complex secretome involved in mollusk shell construction is encoded primarily by rapidly evolving genes.

### Localized expression of mantle genes

**Figure 3:**
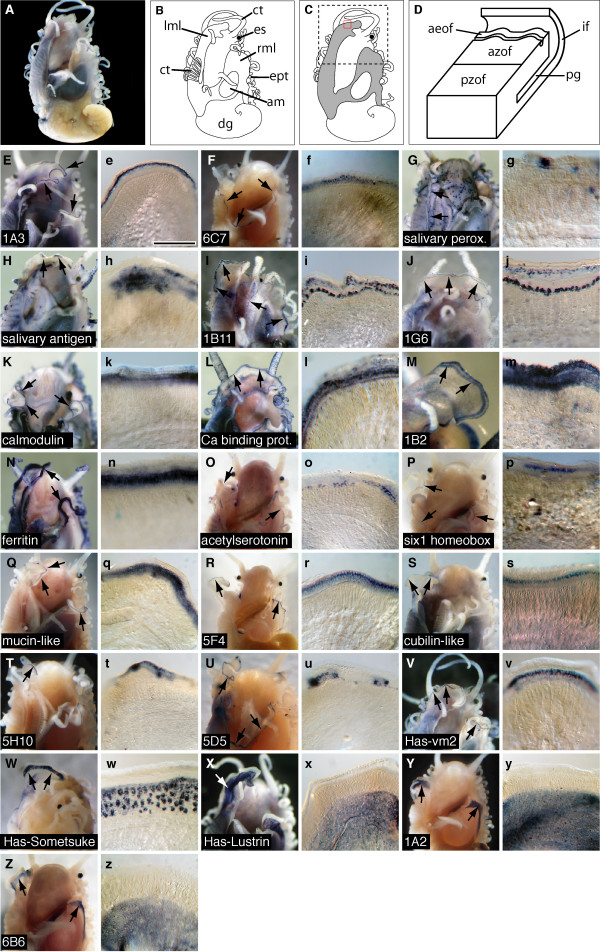
**Modular spatial expression of mantle ESTs**. Localized expression of 22 *H. asinina *ESTs in the juvenile mantle revealed by whole mount *in situ *hybridization (WMISH). (a) A representative de-shelled 5 mm (anterior-posterior) juvenile animal viewed dorsally. (b) Schematic representation of the animal shown in (a). The gills (g), digestive gland (dg), adductor muscle (am), epipodial tentacles (ept), right mantle lobe (rml), eyespot (es), cephalic tentacles (ct) and left mantle lobe (lml) are indicated. (c) The mantle tissue that covers the majority of the dorsal surface of the animal is shaded grey. The region illustrated in each uppercase panel below is indicated by the dashed box, and in each lowercase panel by the red box. (d) A schematic representation of the distal region of the mantle tissue where biomineralization takes place, corresponding to the red box in (c). The posterior zone of the outer fold (pzof), anterior zone of the outer fold (azof), periostracal groove (pg), inner fold (if), and anterior edge of the outer fold (aeof) are indicated. The first panel (uppercase) in each pair is a dorsal view of the anterior end of a juvenile, the second (lowercase) is a magnified view of the dissected mantle edge. Arrows in the first panel of each pair indicate the mantle and representative staining patterns. (E-G) Genes expressed discontinuously within the inner fold of the mantle. (H and h) Gene expressed discontinuously within both the inner mantle fold and the anterior crease of the outer fold. (I-M) Genes expressed continuously within both the inner fold and the anterior crease of the outer fold. (N and n) Gene expressed continuously within the anterior crease of the outer fold. (O-V) Genes expressed discontinuously within the anterior crease of outer fold. (W and w) *HasSom *expressed discontinuously within the anterior zone of the outer fold the mantle. (X-Z) Genes expressed ubiquitously within the posterior zone of the outer fold, corresponding with the area of nacre production. See Additional File 1 for details of ESTs used for *in situ *hybridization. Representative scale bar in (e) (for the second panel in each pair) is 200 μm. All animals possessed shells of between 5 and 6 mm (anterior-posterior).

In order to infer the function of a subset of these ESTs, we selected 22 for spatial expression analysis on the basis of whether they were novel, predicted to be secreted, possessed repetitive domains and/or shared significant similarity with a gene likely to play a role in biomineralization (Figure 3). Of these, 11 are novel and one appears to have a homologue only in the *Lottia *genome (i.e. gastropod-specific; Figure 3M). The spatial expression patterns of these 22 mantle ESTs reveals a diversity of territories and cell types in the mantle (Figure 3), and highlights the modular nature of the mantle tissue [24]. Some of these (9 of 22) are expressed homogenously in one or more zones responsible for the creation of different shell layers. For example, *LustrinA*, first isolated from *Haliotis rufescens*, the gene product of which contributes to the elastomeric properties of the nacreous layer [11,27,47-50], is expressed, along with two other novel genes, in the mantle territory responsible for the production of the inner nacreous layer (Figure 3X–Z). Other evolutionarily conserved genes, including calmodulin and a calcium-binding protein, are expressed continuously along the length of the mantle within both the inner fold and the anterior crease of the outer fold (Figure 3I–L), while others, such as ferritin, are expressed only in the outer fold (Figure 3M, N). The shared spatial expression of ferritin and calmodulin genes between the Bivalvia and Gastropoda suggests they play a conserved role in the formation or modification of the periostracum [34,51]. The remaining 13 genes analyzed here are restricted to specific mantle territories and are expressed discontinuously along the length of the mantle; either in the inner fold alone (Figure 3E–G), the inner mantle fold and the anterior crease of the outer fold (Figure 3H), the anterior crease of outer fold alone (Figure 3O–V), or the anterior zone of the outer fold the mantle (Figure 3W). The dynamic spatial expression of these genes along the length of the mantle, regardless of precise zone, implies that they are contributing to the patterning of shell structure and/or coloration [52,53].

### Blue pigmentation gene

**Figure 4:**
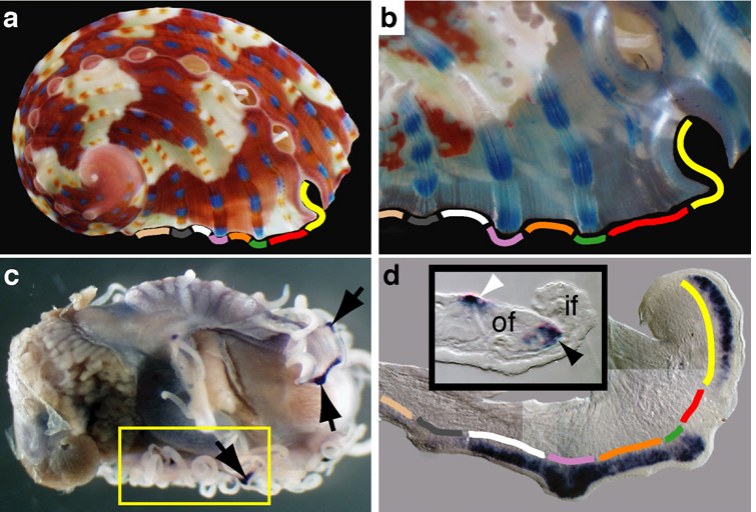
**HasSom expression correlates with shell pigmentation**. (a) Juvenile *H. asinina *shell prior to whole mount *in situ *hybridization against *HasSom*. (b) The same shell partially decalcified. (c) Dorsal view of shell-less juvenile illustrating *HasSom *expression (arrows). (d) Dissected portion of the mantle (corresponding to the boxed region in c). Coloured lines indicate corresponding regions of the shell and periostracum in (a) and (b). A cross section (insert) shows *HasSom *expression restricted to the dorsal surface of the outer fold (white arrowhead), and to the dorsal surface of the periostracal groove (black arrowhead).

*Has-sometsuke *(*HasSom*) is the only gene in this study to be expressed in the anterior zone of the outer fold of the mantle (Figure 3W and 4) and the only gene to map precisely to a shell pigmentation pattern (Figure 4). *HasSom *transcripts are only detected in territories of the mantle that underlie regions of the shell that are producing blue dots and fields of red, which become blue upon de-calcification (Figure 1). Blue dots (Figure 1a) correspond to zones of high *HasSom *expression relative to regions of red (Figure 4). The relative abundance (blue dot or red field) or absence (cream field) of this single gene product is consistent with a role in creating the juvenile shell pattern. Interestingly, BLAST alignments indicate the derived Sometsuke protein shares weak similarity to the ependymins, a family of rapidly evolving extracellular glycoproteins previously only found in deuterostomes ([[54]; Supplementary Figure 1] and Additional File 4). There is currently no evidence of this protein family encoded in ecdysozoan and basal metazoan genomes; we could not detect an ependymin gene within the *Lottia, Nematostella, Amphimedon *or *Hydra *genomes. Ependymin is highly expressed in vertebrate cerebrospinal fluid [55] and may be involved in echinoderm tissue regeneration [56]. With disparate roles in these three phyla and apparent loss from many genomes, it is difficult to infer the ancestral role of this protein in the Bilateria. However, various functional features of this protein, including its ability to bind calcium [57] and to undergo polymerization into insoluble fibrils [55] support a role for Sometsuke in shell construction and patterning.

## Conclusion

The spatial expression profiles of the genes surveyed here support the supposition that specific mantle zones influence the crystal morphology of discrete layers of the mature shell. Underlying these structural differences is a zone-specific secretome. It appears likely that highly dynamic gene expression patterns along the length of a given mantle zone contribute to shell patterning. In the case of juvenile *H. asinina*, these patterns include: the ridge and valley architecture; the periodic formation of respiratory pores, the regular deposition of blue and orange colored dots on the ridges, and the swathes of cream and red/brown fields that cover the shell. The correspondence of *HasSom *expression with shell coloration indicates that there are direct relationships between gene expression and shell patterns, which allows for understanding the molecular basis of structural and color patterning.

The modular design of the molluskan mantle [24,58], along with distinct patterning mechanisms within each zone, allows for immense variation in shell structure and pattern in shell-building mollusks. This morphogenetic system, combined with a complex and rapidly evolving secretome, as revealed here, is likely to have provided the foundation from which the incredible diversity of molluskan shell shapes and patterns has evolved. Despite the advantages of this approach to the rapid identification of novel biomineralizing proteins, it must be pointed out that common post-translational modifications (glycosylation, lipid transfer etc.) that are likely to greatly increase the diversity of the organic matrix, will not be detected by this approach, and further underscores the fact that we are some way from a detailed understanding of how nature generates these functional and beautiful structures.

## Methods

### Library construction, sequencing and EST annotation

A directionally cloned cDNA library was constructed from total RNA extracted from the mantle tissue of ten 7–15 mm juvenile *H. asinina *using a BD Biosciences SMART library construction kit. Phages were converted into plasmid DNA following the manufacturer's instructions and sequenced using ABI chemistry v 3.1 [59]. EST sequences were clustered using ClustalW [60,61] and inspected visually to yield consensus contiguous sequences and a non-redundant collection of ESTs. ESTs were first annotated based on SWISSPROT and NCBI nucleotide, protein and EST database searches at the National Center for Biotechnology Information (NCBI) using the BLAST [62] family of programs [63] and classified according to function [64]. At the time of writing 900,172,457 *L. scutum *traces were available from the NCBI trace archive [65]. These traces were downloaded and searched against our dataset using the standalone BLAST package (TBLASTx and TBLASTn algorithms with default gap costs and the BLOSUM62 matrix). Putative ORFs were identified in ESTs either through sequence similarity or ORF Finder [66] and manual inspection. Among the novel ESTs (those ESTs sharing no significant similarity with publicly available sequences) only ORFs larger than 50 codons, encoded by the positive strand, and beginning with a methionine residue were accepted. All conceptually derived protein sequences were assessed for the presence of a leader sequence using the SignalP 3.0 [67] server [68] and were classified into extracellular or intracellular categories. An EST encoding a novel protein was only accepted as destined for secretion if both SignalP 3.0 algorithms (neural network and hidden Markov model) identified the presence of a signal peptide and a cleavage site, and if the Markov model probability was higher than 90% (in most cases this was >95%). Sequences are deposited at NCBI under accession numbers DW986183 to DW986511. *Has-vm2 *and *Has-lustrin *have accession numbers DQ298397 and DQ298402 respectively.

### *In situ *hybridization, histology and electron microscopy

Juveniles (1–10 mm) of *H. asinina *were relaxed with 1 M MgCl_2 _in seawater and then fixed for 1 h in 4% paraformaldehyde, 0.1 M 3-(*N*-morpholino) propane sulfonic acid pH 7.5, (MOPS), 2 mM MgSO_4_, 1 mM ethyleneglycoltetra-acetic acid (EGTA) and 0.5 M NaCl. Fixed animals were then washed five times with 100% ethanol and stored in 75% ethanol at -20°C. All DIG-labeled riboprobes were produced using either SP6, T3 or T7 RNA polymerase and a PCR amplicon of the desired clone. Several sense controls were performed with probes of various GC contents and lengths to assess background patterns. Endogenous alkaline phosphatase activity of the mantle tissue was also assessed and found not to be present following the in situ procedure. Whole mount *in situ *hybridization (WMISH) was performed as previously described [69] usually at a hybridization temperature of 62°C. Sections (6 μm) were produced by mounting juveniles that had undergone WMISH in EPON 812 and sectioning using a Leica Ultracut T. Prior to WMISH shells were removed by incubation in 1× PBS, 4% paraformaldehyde and 350 mM EDTA. Gene expression within the mantle was correlated to shell patterning activity by photographing individual shells prior to decalcification and relating this to WMISH results.

For transmission electron microscopy, juveniles were relaxed as described above and mantle tissue was dissected and processed according to [70]. Briefly, tissue was prefixed in low osmium (0.05% OsO_4 _in 4% glutaraldehyde, 0.2 M Na cacodylate, 0.1 M NaCl and 0.35 M sucrose, pH 7.2) for 10 min. This was followed with a primary fixation in the same fixative, but lacking osmium, for 1 h. The tissue was then washed twice in buffer (0.3 M NaCl and 0.2 M Na cacodylate, pH 7.2) before a post fixation in osmium (1% OsO_4_, 0.3 M NaCl and 0.2 M Na cacodylate, pH 7.2) for 1 h. Samples were then dehydrated through a graded series of ethanol, embedded in EPON 812 and 60 nm sections taken using a Leica Ultracut T. Sections were stained with uranyl acetate and lead citrate and viewed in a JEOL JEM 1010 transmission electron microscope at 80 kV.

For scanning electron microscopy, whole juveniles with the shell removed were fixed and dehydrated as described above, then infiltrated and dried overnight in hexamethyldisilisane. Juveniles and unfixed shells were mounted on stubs, sputter-coated with platinum and viewed in a JEOL JSM 6300 scanning electron microscope at 15 kV.

## Authors' contributions

DJJ contributed to the conception and design of the project, analysis and interpretation of the data, carried out molecular genetic studies, phylogenetic analyses and drafted the manuscript. CM conducted molecular genetic studies. KG conducted histological and electron microscopy studies. FS contributed to the conception and design of the project and the analysis of EST data. GW contributed to the conception and design of the project and drafted the manuscript. BMD contributed to the conception and design of the project, analysis and interpretation of the data and drafted the manuscript.

## Supplementary Material

Additional File 1 **Table 1: **Complete list of *H. asinina *mantle ESTs and associated TBLASTX and BLASTN results. Click here for file

Additional File 2 **Table 2: **Genes reported to be involved in molluskan biomineralization that were searched against the *Lottia scutum *genome. Click here for file

Additional File 3 **Table 3: ***H. asinina *mantle ESTs and biomineralization genes that share significant similarity with a *Lottia scutum *genomic trace. Click here for file

Additional File 4 **Fig. 5: **Phylogenetic analysis of *Has-sometsuke *(*HasSom*). (a) An unrooted parsimony tree (1000 bootstrap replicates) and (b) an unrooted Bayesian phylogram (200,000 generations, 1,000 burn in trees) illustrate the divergent nature of both *Haliotis asinina *and *Strongylocentrotus purpuratus *ependymin-like sequences from previously reported ependymin sequences. In agreement with a previous phylogenetic analysis [56], the echinoderms *Lytechinus variegatus *and *Holothuria glaberima *form a clade in association with the mammalian and *Xenopus *sequences. The amphioxus and *Strongylocentrotus purpuratus *sequences were not included in this previous analysis. (c) The protein alignment used to generate the trees shown in (a) and (b). Accession numbers are indicated. Alignments were created with both ClustalW and Dialign, compared and manually optimized. Arrows indicate conserved cysteine residues that are characteristic of the ependymin proteins [54]. Red arrow indicates the presence of a cysteine residue only in the *H. asinina, S. purpuratus *and amphioxus sequences. Asterisk indicates the loss of a cysteine residue in *S. purpuratus*. Figures following alignments are percentage identities and percentage positives respectively and were generated by significant pairwise bl2seq alignments between *HasSom *and each individual sequence. Click here for file

## References

[B1] Dove PM, De Yoreo JJ, Weiber S, (Eds) (2004). Biomineralization.

[B2] Marshall CR (2005). Nomothetism and understanding the Cambrian "explosion". Palaios.

[B3] Knoll AH (2003). Biomineralization and evolutionary history. Rev Mineral Geochem.

[B4] Morris SC (2000). The Cambrian "explosion": slow-fuse or megatonnage?. Proc Natl Acad Sci USA.

[B5] Porter SM, Meisterfeld R, Knoll AH (2003). Vase-shaped microfossils from the Neoproterozoic Chuar group, Grand Canyon: a classification guided by modern testate amoebae. J Paleontology.

[B6] Kirschvink JL, Hagadorn JW, Weinheim BE (2000). A grand unified theory of biomineralization. The Biomineralisation of Nano- and Micro-Structures.

[B7] Marin F, Luquet G (2004). Molluscan shell proteins. Comptes Rendus Palevol.

[B8] Wilt FH, Killian CE, Livingston BT (2003). Development of calcareous skeletal elements in invertebrates. Differentiation.

[B9] Wilt FH (2005). Developmental biology meets materials science: morphogenesis of biomineralized structures. Dev Biol.

[B10] Weiner S, Sagi I, Addadi L (2005). Choosing the crystallization path less traveled. Science.

[B11] Shen XY, Belcher AM, Hansma PK, Stucky GD, Morse DE (1997). Molecular cloning and characterization of lustrin A, a matrix protein from shell and pearl nacre of Haliotis rufescens. J Biol Chem.

[B12] Duplat D, Puissegur M, Bedouet L, Rousseau M, Boulzaguet H, Milet C, Sellos D, Van Wormhoudt A, Lopez E (2006). Identification of calconectin, a calcium-binding protein specifically expressed by the mantle of *Pinctada margaritifera*. FEBS Lett.

[B13] Weiss IM, Gohring W, Fritz M, Mann K (2001). Perlustrin, a *Haliotis laevigata *(Abalone) nacre protein, is homologous to the insulin-like growth factor binding protein N-terminal module of vertebrates. Biochem Biophys Res Comm.

[B14] Mann K, Weiss IM, Andre S, Gabius H-J, Fritz M (2000). The amino-acid sequence of the abalone (*Haliotis laevigata*) nacre protein perlucin. Detection of a functional C-type lectin domain with galactose/mannose specificity. Eur J Biochem.

[B15] Miyamoto H, Miyashita T, Okushima M, Nakano S, Morita T, Matsushiro A (1996). A carbonic anhydrase from the nacreous layer in oyster pearls. Proc Natl Acad Sci USA.

[B16] Samata T, Hayashib N, Konoa M, Hasegawac K, Horitad C, Akerad S (1999). A new matrix protein family related to the nacreous layer formation of *Pinctada fucata*. FEBS Lett.

[B17] Addadi L, Joester D, Nudelman F, Weiner S (2006). Mollusk shell formation: a source of new concepts for understanding biomineralization processes. Chemistry – A European Journal.

[B18] Lowenstam HA, Weiner S, (Eds) (1989). On Biomineralization.

[B19] Weiner S, Traub W (1984). Macromolecules in mollusk shells and their functions in biomineralization. Phil Trans Royal Soc London B- Biol Sci.

[B20] Wainwright SA (1969). Stress and design in bivalved mollusc shell. Nature.

[B21] Kniprath E (1981). Ontogeny of the molluscan shell field – a review. Zoologica Scripta.

[B22] Brusca RC, Brusca GJ (2002). Invertebrates.

[B23] Saleuddin ASM, Petit HP, Saleuddin ASM, Wilbur KM (1983). The mode of formation and the structure of the periostracum. The Mollusca.

[B24] Jolly C, Berland S, Milet C, Borzeix S, Lopez E, Doumenc D (2004). Zonal localization of shell matrix proteins in mantle of *Haliotis tuberculata *(Mollusca, Gastropoda). Mar Biotech.

[B25] Saleuddin ASM (1974). An electron microscopic study of the formation and structure of the periostracum in Astarte (Bivalvia). Can J Zoology.

[B26] Bevelander G, Nakahara H (1970). An electron microscope study of the formation and structure of the periostracum of a gastropod, *Littorina littorea*. Calc Tiss Int.

[B27] Zhang B, Wustman BA, Morse D, Evans JS (2002). Model peptide studies of sequence regions in the elastomeric biomineralization protein, lustrin A. I. The C-domain consensus-PG-, -NVNCT-motif. Biopolymers.

[B28] Thompson JB, Paloczi GT, Kindt JH, Michenfelder M, Smith BL, Stucky G, Morse DE, Hansma PK (2000). Direct observation of the transition from calcite to aragonite growth as induced by abalone shell proteins. Biophys J.

[B29] Falini G, Albeck S, Weiner S, Addadi L (1996). Control of aragonite or calcite polymorphism by mollusk shell macromolecules. Science.

[B30] Zaremba CM, Belcher AM, Fritz M, Li YL, Mann S, Hansma PK, Morse DE, Speck JS, Stucky GD (1996). Critical transitions in the biofabrication of abalone shells and flat pearls. Chem Mater.

[B31] Gotliv BA, Addadi L, Weiner S (2003). Mollusk shell acidic proteins: in search of individual functions. Chem Biochem.

[B32] Jackson D, Williams KC, Degnan BM (2001). Suitability of Australian formulated diets for aquaculture of the tropical abalone *Haliotis asinina *Linnaeus. J Shellfish Res.

[B33] Li S, Xie LP, Zhang C, Zhang Y, Gu MZ, Zhang RQ (2004). Cloning and expression of a pivotal calcium metabolism regulator: calmodulin involved in shell formation from pearl oyster (*Pinctada fucata*). Comp Biochem Physiol B.

[B34] Zhang Y, Meng QX, Jiang TM, Wang HZ, Xie LP, Zhang RQ (2003). A novel ferritin subunit involved in shell formation from the pearl oyster (*Pinctada fucata*). Comparative Biochemistry and Physiology B.

[B35] Grimmond SM, Miranda KC, Yuan Z, Davis MJ, Hume DA, Yagi K, Tominaga N, Bono H, Hayashizaki Y, Okazaki Y (2003). The mouse secretome: functional classification of the proteins secreted into the extracellular environment. Gen Res.

[B36] Klee EW, Carlson DF, Fahrenkrug SC, Ekker SC, Ellis LBM (2004). Identifying secretomes in people, pufferfish and pigs. Nucleic Acids Research.

[B37] Clark HF, Gurney AL, Abaya E, Baker K, Baldwin D, Brush J, Chen J, Chow B, Chui C, Crowley C (2003). The Secreted Protein Discovery Initiative (SPDI), a large-scale effort to identify novel human secreted and transmembrane proteins: A bioinformatics assessment. Gen Res.

[B38] Katti KS, Katti DR, Pradhan SM, Bhosle A (2005). Platelet interlocks are the key to toughness and strength in nacre. J Mat Res.

[B39] Nassif N, Pinna N, Gehrke N, Antonietti M, Jager C, Colfen H (2005). Amorphous layer around aragonite platelets in nacre. Proc Natl Acad Sci USA.

[B40] Lin A, Meyers MA (2005). Growth and structure in abalone shell. Mat Sci Eng A.

[B41] Fu G, Valiyaveettil S, Wopenka B, Morse DE (2005). CaCO_3 _biomineralization: acidic 8-kDa proteins isolated from aragonitic abalone shell nacre can specifically modify calcite crystal morphology. Biomacromolecules.

[B42] van Beek JD, Hess S, Vollrath F, Meier BH (2002). The molecular structure of spider dragline silk: folding and orientation of the protein backbone. Proc Natl Acad Sci USA.

[B43] Wilson RC, Long F, Maruoka EM, Cooper JB (1994). A new proline-rich early nodulin from *Medicago truncatula *is highly expressed in nodule meristematic cells. Plant Cell.

[B44] JGI sequencing plans. http://www.jgi.doe.gov/sequencing/cspseqplans.html.

[B45] Weiss IM, Kaufmann S, Mann K, Fritz M (2000). Purification and characterization of Perlucin and Perlustrin, two new proteins from the shell of the mollusc *Haliotis laevigata*. Biochem Biophys Res Comm.

[B46] Marin F, Corstjens P, de Gaulejac B, de Vrind-De Jong E, Westbroek P (2000). Mucins and molluscan calcification. Molecular characterization of mucoperlin, a novel mucin-like protein from the nacreous shell layer of the fan mussel *Pinna nobilis *(Bivalvia, Pteriomorphia). J Bio Chem.

[B47] Wustman BA, Weaver JC, Morse DE, Evans JS (2003). Characterization of a Ca(II)-, mineral-interactive polyelectrolyte sequence from the adhesive elastomeric biomineralization protein lustrin A. Langmuir.

[B48] Wustman BA, Morse DE, Evans JS (2002). Structural analyses of polyelectrolyte sequence domains within the adhesive elastomeric biomineralization protein Lustrin A. Langmuir.

[B49] Smith BL, Schaffer TE, Viani M, Thompson JB, Frederick NA, Kindt J, Belcher A, Stucky GD, Morse DE, Hansma PK (1999). Molecular mechanistic origin of the toughness of natural adhesives, fibres and composites. Nature.

[B50] Wustman BA, Weaver JC, Morse DE, Evans JS (2003). Structure-function studies of the Lustrin A polyelectrolyte domains, RKSY and D4. Conn Tiss Res.

[B51] Li S, Xie LP, Ma ZJ, Zhang RQ (2005). cDNA cloning and characterization of a novel calmodulin-like protein from pearl oyster *Pinctada fucata*. FEBS J.

[B52] Meinhardt H, Prusinkiewicz P, Fowler DR (1995). The Algorithmic Beauty of Sea Shells.

[B53] Meinhardt H, Klinger M (1987). A model for pattern formation on the shells of molluscs. J Theoretical Biol.

[B54] Orti G, Meyer A (1996). Molecular evolution of ependymin and the phylogenetic resolution of early divergences among euteleost fishes. Molecular Biology and Evolution.

[B55] Shashoua VE (1988). Monomeric and polymeric forms of ependymin: a brain extracellular glycoprotein implicated in memory consolidation processes. Neurochem Res.

[B56] Suarez-Castillo EC, Medina-Ortiz WE, Roig-Lopez JL, Garcia-Arraras JE (2004). Ependymin, a gene involved in regeneration and neuroplasticity in vertebrates, is overexpressed during regeneration in the echinoderm *Holothuria glaberrima*. Gene.

[B57] Ganss B, Hoffmann W (1993). Calcium-binding to sialic acids and Its effect on the conformation of ependymins. Eur J Biochem.

[B58] Winther RG (2001). Varieties of modules: kinds, levels, origins, and behaviors. J Exp Biol.

[B59] Sambrook J, Russell DW (2001). Molecular cloning: a laboratory manual.

[B60] Chenna R, Sugawara H, Koike T, Lopez R, Gibson TJ, Higgins DG, Thompson JD (2003). Multiple sequence alignment with the Clustal series of programs. Nucleic Acids Res.

[B61] ClustalW. http://www.ebi.ac.uk/clustalw/index.html.

[B62] Altschul SF, Madden TL, Schaffer AA, Zhang J, Zhang Z, Miller W, Lipman DJ (1997). Gapped BLAST and PSI-BLAST: a new generation of protein database search programs. Nucleic Acids Res.

[B63] BLAST. http://www.ncbi.nlm.nih.gov/BLAST/.

[B64] Lee Y-H, Huang GM, Cameron RA, Graham G, Davidson EH, Hood L, Britten RJ (1999). EST analysis of gene expression in early cleavage-stage urchin embryos. Development.

[B65] Trace archive. http://www.ncbi.nlm.nih.gov/Traces/trace.cgi?.

[B66] ORF finder. http://www.ncbi.nlm.nih.gov/gorf/orfig.cgi.

[B67] Dyrlov Bendtsen J, Nielsen H, von Heijne G, Brunak S (2004). Improved prediction of signal peptides: SignalP 3.0. J Mol Biol.

[B68] SignalP 3.0. http://www.cbs.dtu.dk/services/SignalP/.

[B69] Giusti AF, Hinman VF, Degnan SM, Degnan BM, Morse DE (2000). Expression of a Scr/Hox5 gene in the larval central nervous system of the gastropod Haliotis, a non-segmented spiralian lophotrochozoan. Evol Dev.

[B70] Eisenman EA, Alfert M (1982). A new fixation procedure for preserving the ultrastructure of marine invertebrate tissues. J Microscopy.

