# Supplementary material for: A rapidly evolving secretome builds and patterns a sea shell
Source: BMC Biol. 2006 Nov 22;4:40. doi: 10.1186/1741-7007-4-40 (PMC1676022; doi:10.1186/1741-7007-4-40)
Supplement: Additional File 1 — Table 1: Complete list of H. asinina mantle ESTs and associated TBLASTX and BLASTN results. [file 1741-7007-4-40-S1.doc]

**Additional Table 1. Complete list of *H. asinina* mantle ESTs and associated TBLASTX and BLASTN results.**

| **Clone ID** | **Brief descriptor** | **GenBank accession number** | **E value** | **Category** |
| --- | --- | --- | --- | --- |
| Mantle library clone 1A1 | Novel H. asinina mantle EST 1 | DW986183 | x | Novel (Intracellular) |
| Mantle library clone 1A10 | Novel H. asinina mantle EST 3 | DW986184 | x | Novel (Intracellular) |
| Mantle library clone 1A4 | 18S rRNA / Proline rich | DW986185 | 1 e-176 | Intracellular with a GenBank homologue |
| Mantle library clone 1A5 | Novel H. asinina mantle EST 19 | DW986186 | x | Novel (Leader sequence detected) |
| Mantle library clone 1A6 | thioredoxin-like 5 | DW986187 | 9 e-35 | Novel (Intracellular) |
| Mantle library clone 1A7 | ribosomal protein S30 | DW986188 | 8 e-46 | Intracellular with a GenBank homologue |
| Mantle library clone 1A8 | cytochrome b | DW986189 | 3 e-85 | Intracellular with a GenBank homologue |
| Mantle library clone 1A9 | Novel H. asinina mantle EST 23 | DW986190 | x | Novel (Intracellular) |
| Mantle library clone 1B1 | hypothetical protein | DW986191 | 3 e-45 | Novel (Intracellular) |
| Mantle library clone 1B10 | Novel H. asinina mantle EST 26 | DW986192 | x | Novel (Intracellular) |
| Mantle library clone 1B12 | RhoA | DW986193 | 2 e-60 | Trafficking |
| Mantle library clone 1B2* | Novel H. asinina mantle EST 30 | DW986194 | x | Novel (Leader sequence detected) |
| Mantle library clone 1B3 | Novel H. asinina mantle EST 33 | DW986195 | x | Novel (Intracellular) |
| Mantle library clone 1B4 | hypothetical protein | DW986196 | 6 e-27 | Novel (Intracellular) |
| Mantle library clone 1B6 | similar to novel cell death-regulatory protein GRIM19 | DW986197 | 2 e-27 | Intracellular with a GenBank homologue |
| Mantle library clone 1B7 | ribosomal protein L27 | DW986198 | 3 e-67 | Intracellular with a GenBank homologue |
| Mantle library clone 1B8 | Novel H. asinina mantle EST 40 | DW986199 | x | Novel (Intracellular) |
| Mantle library clone 1C1 | Novel H. asinina mantle EST 44 | DW986200 | x | Novel (Intracellular) |
| Mantle library clone 1C10 | ribosomal protein L10 | DW986201 | 2 e-50 | Intracellular with a GenBank homologue |
| Mantle library clone 1C2 | Novel H. asinina mantle EST 50 | DW986202 | x | Novel (Intracellular) |
| Mantle library clone 1C5 | Novel H. asinina mantle EST 57 | DW986203 | x | Novel (Leader sequence detected) |
| Mantle library clone 1C6 | Novel H. asinina mantle EST 61 | DW986204 | x | Novel (Intracellular) |
| Mantle library clone 1C7 | hypothetical protein | DW986205 | 2 e-9 | Novel (Intracellular) |
| Mantle library clone 1C8 | hypothetical protein | DW986206 | 2 e-57 | Novel (Intracellular) |
| Mantle library clone 1D1 | Novel H. asinina mantle EST 70 | DW986207 | x | Novel (Leader sequence detected) |
| Mantle library clone 1D10 | Novel H. asinina mantle EST 71 | DW986208 | x | Novel (Intracellular) |
| Mantle library clone 1D2 | Novel H. asinina mantle EST 78 | DW986209 | x | Novel (Intracellular) |
| Mantle library clone 1D3 | Novel H. asinina mantle EST 79 | DW986210 | x | Novel (Intracellular) |
| Mantle library clone 1D4 | Xtld protein | DW986211 | 1 e-4 | Novel (Leader sequence detected) |
| Mantle library clone 1D5 | Novel H. asinina mantle EST 81 | DW986212 | x | Novel (Intracellular) |
| Mantle library clone 1D6 | Novel H. asinina mantle EST 85 | DW986213 | x | Novel (Intracellular) |
| Mantle library clone 1D7* | salivary secreted antigen-5 precursor | DW986214 | 5 e -10 | Leader sequence detected with GenBank homologue |
| Mantle library clone 1D9 | Novel H. asinina mantle EST 91 | DW986215 | x | Novel (Intracellular) |
| Mantle library clone 1E3 | Novel H. asinina mantle EST 103 | DW986216 | x | Novel (Leader sequence detected) |
| Mantle library clone 1E4* | 16 Kda calcium binding protein | DW986217 | 4 e-10 | Trafficking |
| Mantle library clone 1E5 | Novel H. asinina mantle EST 108 | DW986218 | x | Novel (Intracellular) |
| Mantle library clone 1E6* | Sometsuke | DW986219 | x | Novel (Leader sequence detected) |
| Mantle library clone 1E7 | cysteine proteinase inhibitor | DW986220 | 1 e-22 | Intracellular with a GenBank homologue |
| Mantle library clone 1E8 | clathrin light polypeptide | DW986221 | 2 e-9 | Trafficking |
| Mantle library clone 1E9 | hemocyanin | DW986222 | 6 e-10 | Intracellular with a GenBank homologue |
| Mantle library clone 1F2 | Novel H. asinina mantle EST 124 | DW986223 | x | Novel (Intracellular) |
| Mantle library clone 1F3 | Novel H. asinina mantle EST 126 | DW986224 | x | Novel (Leader sequence detected) |
| Mantle library clone 1F4 | Novel H. asinina mantle EST 129 | DW986225 | x | Novel (Leader sequence detected) |
| Mantle library clone 1F7 | Novel H. asinina mantle EST 139 | DW986226 | x | Novel (Intracellular) |
| Mantle library clone 1F8 | Novel H. asinina mantle EST 140 | DW986227 | x | Novel (Intracellular) |
| Mantle library clone 1G12 | ribosomal protein L19 | DW986228 | 4 e-43 | Intracellular with a GenBank homologue |
| Mantle library clone 1G2 | actin-depolymerizing factor | DW986229 | 4 e-17 | Intracellular with a GenBank homologue |
| Mantle library clone 1G3 | ribosomal protein L17 | DW986230 | 3 e-81 | Intracellular with a GenBank homologue |
| Mantle library clone 1G5 | Novel H. asinina mantle EST 157 | DW986231 | x | Novel (Intracellular) |
| Mantle library clone 1H2 | inhibitor of apoptosis protein 3 | DW986232 | 1 e-22 | Intracellular with a GenBank homologue |
| Mantle library clone 1H5 | 40S ribosomal protein S9 | DW986233 | 1 e-100 | Intracellular with a GenBank homologue |
| Mantle library clone 1H7* | salivary peroxidase | DW986234 | 1 e-10 | Leader sequence detected with GenBank homologue |
| Mantle library clone 1H8 | NADH dehydrogenase | DW986235 | 2 e-25 | Intracellular with a GenBank homologue |
| Mantle library clone 3A10 | Novel H. asinina mantle EST 4 | DW986236 | x | Novel (Intracellular) |
| Mantle library clone 3A11 | Novel H. asinina mantle EST 7 | DW986237 | x | Novel (Leader sequence detected) |
| Mantle library clone 3A4 | Novel H. asinina mantle EST 15 | DW986238 | x | Novel (Intracellular) |
| Mantle library clone 3A5 | Novel H. asinina mantle EST 20 | DW986239 | x | Novel (Intracellular) |
| Mantle library clone 3A7 | Novel H. asinina mantle EST 21 | DW986240 | x | Novel (Intracellular) |
| Mantle library clone 3B1 | Jun dimerization protein | DW986241 | 3 e -6 | Transcription factor |
| Mantle library clone 3B2 | Ornithine decarboxylase antizyme | DW986242 | 4 e -26 | Intracellular with a GenBank homologue |
| Mantle library clone 3B3 | Novel H. asinina mantle EST 34 | DW986243 | x | Novel (Intracellular) |
| Mantle library clone 3B8 | Novel H. asinina mantle EST 41 | DW986244 | x | Novel (Intracellular) |
| Mantle library clone 3C1 | translationally controlled tumor protein | DW986245 | 4 e -14 | Intracellular with a GenBank homologue |
| Mantle library clone 3C10 | Novel H. asinina mantle EST 45 | DW986246 | x | Novel (Intracellular) |
| Mantle library clone 3C12 | Scavenger receptor class F | DW986247 | 2 e -8 | Leader sequence detected with GenBank homologue |
| Mantle library clone 3C2 | Novel H. asinina mantle EST 51 | DW986248 | x | Novel (Leader sequence detected) |
| Mantle library clone 3C4 | Novel H. asinina mantle EST 54 | DW986249 | x | Novel (Intracellular) |
| Mantle library clone 3C9 | ribosomal protein L18 | DW986250 | 1 e -69 | Intracellular with a GenBank homologue |
| Mantle library clone 3D1 | ribosomal protein S13 | DW986251 | 8 e -76 | Intracellular with a GenBank homologue |
| Mantle library clone 3D2 | 40S ribosomal protein S23 | DW986252 | 3 e -72 | Intracellular with a GenBank homologue |
| Mantle library clone 3D4 | ink toxin 3 | DW986253 | 8 e -7 | Leader sequence detected with GenBank homologue |
| Mantle library clone 3D8 | Novel H. asinina mantle EST 88 | DW986254 | x | Novel (Intracellular) |
| Mantle library clone 3E6 | ribosomal protein L7A | DW986255 | 2 e -74 | Intracellular with a GenBank homologue |
| Mantle library clone 3E9 | Novel H. asinina mantle EST 117 | DW986256 | x | Novel (Leader sequence detected) |
| Mantle library clone 3F11 | ribosomal protein S15 | DW986257 | 1 e -44 | Intracellular with a GenBank homologue |
| Mantle library clone 3F2 | eukaryotic initiation factor | DW986258 | 6 e -53 | Intracellular with a GenBank homologue |
| Mantle library clone 3F4 | Novel H. asinina mantle EST 130 | DW986259 | x | Novel (Intracellular) |
| Mantle library clone 3F5 | Novel H. asinina mantle EST 135 | DW986260 | x | Novel (Leader sequence detected) |
| Mantle library clone 3F8 | serine/arginine repetitive matrix 2 | DW986261 | 2 e -45 | Intracellular with a GenBank homologue |
| Mantle library clone 3G3 | similar to dpy-30-like protein | DW986262 | 2 e -24 | Intracellular with a GenBank homologue |
| Mantle library clone 3G6 | Novel H. asinina mantle EST 160 | DW986263 | x | Novel (Intracellular) |
| Mantle library clone 3H10 | Novel H. asinina mantle EST 165 | DW986264 | x | Novel (Leader sequence detected) |
| Mantle library clone 3H6 | Novel H. asinina mantle EST 179 | DW986265 | x | Novel (Leader sequence detected) |
| Mantle library clone 3H9 | NADH dehydrogenase | DW986266 | 7 e -18 | Intracellular with a GenBank homologue |
| Mantle library clone 4A2 | ATPase synthase | DW986267 | 4 e -30 | Intracellular with a GenBank homologue |
| Mantle library clone 4A5 | ribosomal protein S3a | DW986268 | 2 e -61 | Intracellular with a GenBank homologue |
| Mantle library clone 4B2 | Novel H. asinina mantle EST 31 | DW986269 | x | Novel (Intracellular) |
| Mantle library clone 4B3 | step II splicing factor | DW986270 | 3 e -26 | Intracellular with a GenBank homologue |
| Mantle library clone 4B4 | salivary lysozyme | DW986271 | 2 e -22 | Leader sequence detected with GenBank homologue |
| Mantle library clone 4B5 | Novel H. asinina mantle EST 37 | DW986272 | x | Novel (Intracellular) |
| Mantle library clone 4B7 | ribosomal protein S27-1 | DW986273 | 6 e -42 | Intracellular with a GenBank homologue |
| Mantle library clone 4C5 | Novel H. asinina mantle EST 58 | DW986274 | x | Novel (Leader sequence detected) |
| Mantle library clone 4D4 | Novel H. asinina mantle EST 80 | DW986275 | x | Novel (Leader sequence detected) |
| Mantle library clone 4D5 | Novel H. asinina mantle EST 82 | DW986276 | x | Novel (Intracellular) |
| Mantle library clone 4E2 | Novel H. asinina mantle EST 100 | DW986277 | x | Novel (Intracellular) |
| Mantle library clone 4E3 | Novel H. asinina mantle EST 104 | DW986278 | x | Novel (Intracellular) |
| Mantle library clone 4E4 | Novel H. asinina mantle EST 105 | DW986279 | x | Novel (Intracellular) |
| Mantle library clone 4E6 | Novel H. asinina mantle EST 112 | DW986280 | x | Novel (Intracellular) |
| Mantle library clone 4F3 | cytochrome c oxidase | DW986281 | 3 e -55 | Intracellular with a GenBank homologue |
| Mantle library clone 4F4 | Novel H. asinina mantle EST 131 | DW986282 | x | Novel (Intracellular) |
| Mantle library clone 4G1 | JunDLa | DW986283 | 3 e -13 | Transcription factor |
| Mantle library clone 5A11 | Novel H. asinina mantle EST 8 | DW986284 | x | Novel (Leader sequence detected) |
| Mantle library clone 5A2 | retrotansposon-like | DW986285 | 4 e-22 | Intracellular with a GenBank homologue |
| Mantle library clone 5A3 | Novel H. asinina mantle EST 13 | DW986286 | x | Novel (Intracellular) |
| Mantle library clone 5A4 | Novel H. asinina mantle EST 16 | DW986287 | x | Novel (Intracellular) |
| Mantle library clone 5A6 | hypothetical protein | DW986288 | 1 e-49 | Novel (Intracellular) |
| Mantle library clone 5A7 | Novel H. asinina mantle EST 22 | DW986289 | x | Novel (Leader sequence detected) |
| Mantle library clone 5B1 | ribosomal protein S14 | DW986290 | 1 e-75 | Intracellular with a GenBank homologue |
| Mantle library clone 5B12 | ribosomal protein S12 | DW986291 | 8 e-75 | Intracellular with a GenBank homologue |
| Mantle library clone 5B2* | Six1 homoeobox protein | DW986292 | 3 e -48 | Transcription factor |
| Mantle library clone 5B3 | solute carrier family 6 | DW986293 | 3 e-54 | Trafficking |
| Mantle library clone 5B4 | ribosomal protein L36 | DW986294 | 2 e-31 | Intracellular with a GenBank homologue |
| Mantle library clone 5B7 | solute carrier family 39 | DW986295 | 2 e-38 | Trafficking |
| Mantle library clone 5B8 | Novel H. asinina mantle EST 42 | DW986296 | x | Novel (Leader sequence detected) |
| Mantle library clone 5C2 | proteasome | DW986297 | 1 e-136 | Intracellular with a GenBank homologue |
| Mantle library clone 5C3 | Novel H. asinina mantle EST 52 | DW986298 | x | Novel (Leader sequence detected) |
| Mantle library clone 5C4 | hypothetical protein | DW986299 | 1 e-7 | Novel (Intracellular) |
| Mantle library clone 5C6 | Novel H. asinina mantle EST 62 | DW986300 | x | Novel (Intracellular) |
| Mantle library clone 5C8 | Novel H. asinina mantle EST 66 | DW986301 | x | Novel (Intracellular) |
| Mantle library clone 5C9 | Novel H. asinina mantle EST 68 | DW986302 | x | Novel (Intracellular) |
| Mantle library clone 5D1* | mucin-like peritrophin | DW986303 | 2 e-7 | Trafficking |
| Mantle library clone 5D10 | Novel H. asinina mantle EST 72 | DW986304 | x | Novel (Leader sequence detected) |
| Mantle library clone 5D11 | Novel H. asinina mantle EST 74 | DW986305 | x | Novel (Intracellular) |
| Mantle library clone 5D2 | thioredoxin reductase | DW986306 | 5 e-76 | Intracellular with a GenBank homologue |
| Mantle library clone 5D3 | ribosomal protein S17 | DW986307 | 1 e-70 | Intracellular with a GenBank homologue |
| Mantle library clone 5D4 | prohibitin | DW986308 | 1 e-120 | Leader sequence detected with GenBank homologue |
| Mantle library clone 5D5* | Novel H. asinina mantle EST 83 | DW986309 | x | Novel (Leader sequence detected) |
| Mantle library clone 5D8 | Novel H. asinina mantle EST 89 | DW986310 | x | Novel (Leader sequence detected) |
| Mantle library clone 5D9 | Novel H. asinina mantle EST 92 | DW986311 | x | Novel (Intracellular) |
| Mantle library clone 5E1 | mitochondrial ADP/ATP translocator protein | DW986312 | 1 e-118 | Intracellular with a GenBank homologue |
| Mantle library clone 5E10 | Novel H. asinina mantle EST 96 | DW986313 | x | Novel (Leader sequence detected) |
| Mantle library clone 5E11 | organic anion transporter | DW986314 | 1 e-12 | Trafficking |
| Mantle library clone 5E5 | Novel H. asinina mantle EST 109 | DW986315 | x | Novel (Intracellular) |
| Mantle library clone 5E8 | ribosomal protein L18a | DW986316 | 3 e-77 | Intracellular with a GenBank homologue |
| Mantle library clone 5F11* | Maf/acetylserotonin O-methyltransferase-like | DW986317 | 1 e-56 | Intracellular with a GenBank homologue |
| Mantle library clone 5F3 | hypothetical protein | DW986318 | 3 e-7 | Novel (Leader sequence detected) |
| Mantle library clone 5F4* | Novel H. asinina mantle EST 132 | DW986319 | x | Novel (Leader sequence detected) |
| Mantle library clone 5F5 | Novel H. asinina mantle EST 136 | DW986320 | x | Novel (Leader sequence detected) |
| Mantle library clone 5F7 | organic solute transporter | DW986321 | 2 e-22 | Trafficking |
| Mantle library clone 5F8 | Novel H. asinina mantle EST 141 | DW986322 | x | Novel (Intracellular) |
| Mantle library clone 5F9 | Novel H. asinina mantle EST 143 | DW986323 | x | Novel (Intracellular) |
| Mantle library clone 5G1 | glyceraldehyde-3-phosphate dehydrogenase | DW986324 | 1 e-133 | Intracellular with a GenBank homologue |
| Mantle library clone 5G10 | Novel H. asinina mantle EST 149 | DW986325 | x | Novel (Intracellular) |
| Mantle library clone 5G12 | Novel H. asinina mantle EST 152 | DW986326 | x | Novel (Intracellular) |
| Mantle library clone 5G3 | CCAAT/enhancer binding protein -1 | DW986327 | 8 e-23 | Transcription factor |
| Mantle library clone 5G4 | Novel H. asinina mantle EST 156 | DW986328 | x | Novel (Intracellular) |
| Mantle library clone 5G5 | Novel H. asinina mantle EST 158 | DW986329 | x | Novel (Leader sequence detected) |
| Mantle library clone 5G6 | ETS-family transcription factor | DW986330 | 9 e-9 | Transcription factor |
| Mantle library clone 5G8 | Novel H. asinina mantle EST 162 | DW986331 | x | Novel (Leader sequence detected) |
| Mantle library clone 5H1 | 18S ribosomal RNA gene | DW986332 | 1 e-158 | Intracellular with a GenBank homologue |
| Mantle library clone 5H10* | Novel H. asinina mantle EST 166 | DW986333 | x | Novel (Leader sequence detected) |
| Mantle library clone 5H11 | Novel H. asinina mantle EST 169 | DW986334 | x | Novel (Intracellular) |
| Mantle library clone 5H2 | metallothionein | DW986335 | 1 e-14 | Trafficking |
| Mantle library clone 5H3 | Novel H. asinina mantle EST 174 | DW986336 | x | Novel (Intracellular) |
| Mantle library clone 5H4 | hypothetical protein | DW986337 | 1 e -119 | Novel (Intracellular) |
| Mantle library clone 5H7 | macrophage expressed protein | DW986338 | 7 e-7 | Leader sequence detected with GenBank homologue |
| Mantle library clone 5H8 | Novel H. asinina mantle EST 180 | DW986339 | x | Novel (Leader sequence detected) |
| Mantle library clone 5H9 | heat shock 70 protein | DW986340 | 2 e-92 | Intracellular with a GenBank homologue |
| Mantle library clone 6A1 | Novel H. asinina mantle EST 2 | DW986341 | x | Novel (Intracellular) |
| Mantle library clone 6A10**(1A2) | Novel H. asinina mantle EST 5 | DW986342 | x | Novel (Leader sequence detected) |
| Mantle library clone 6A11 | Novel H. asinina mantle EST 9 | DW986343 | x | Novel (Leader sequence detected) |
| Mantle library clone 6A3 | Novel H. asinina mantle EST 14 | DW986344 | x | Novel (Intracellular) |
| Mantle library clone 6A4 | Novel H. asinina mantle EST 17 | DW986345 | x | Novel (Intracellular) |
| Mantle library clone 6A6 | ribosomal protein S4 | DW986346 | 1 e-148 | Intracellular with a GenBank homologue |
| Mantle library clone 6A8 | HASP-like protein | DW986347 | 2 e-6 | Intracellular with a GenBank homologue |
| Mantle library clone 6B10 | NOB1 RNA binding protein | DW986348 | 1 e -97 | Novel (Intracellular) |
| Mantle library clone 6B11 | Novel H. asinina mantle EST 27 | DW986349 | x | Novel (Leader sequence detected) |
| Mantle library clone 6B12 | protein disulfide isomerase | DW986350 | 3 e-88 | Intracellular with a GenBank homologue |
| Mantle library clone 6B2 | Novel H. asinina mantle EST 32 | DW986351 | x | Novel (Intracellular) |
| Mantle library clone 6B6* | Novel H. asinina mantle EST 38 | DW986352 | x | Novel (Intracellular) |
| Mantle library clone 6B8 | signal sequence receptor | DW986353 | 8 e-82 | Leader sequence detected with GenBank homologue |
| Mantle library clone 6C10 | Novel H. asinina mantle EST 46 | DW986354 | x | Novel (Leader sequence detected) |
| Mantle library clone 6C11 | protease inhibitor | DW986355 | 5 e-15 | Leader sequence detected with GenBank homologue |
| Mantle library clone 6C12 | Novel H. asinina mantle EST 48 | DW986356 | x | Novel (Intracellular) |
| Mantle library clone 6C4 | Novel H. asinina mantle EST 55 | DW986357 | x | Novel (Intracellular) |
| Mantle library clone 6C5**(1A3) | Novel H. asinina mantle EST 59 | DW986358 | x | Novel (Leader sequence detected) |
| Mantle library clone 6C6 | Novel H. asinina mantle EST 63 | DW986359 | x | Novel (Intracellular) |
| Mantle library clone 6C7* | Novel H. asinina mantle EST 64 | DW986360 | x | Novel (Leader sequence detected) |
| Mantle library clone 6C8 | hemicentin-2 | DW986361 | 9 e-12 | No leader sequence but extracellular |
| Mantle library clone 6C9 | Novel H. asinina mantle EST 69 | DW986362 | x | Novel (Leader sequence detected) |
| Mantle library clone 6D11 | hypothetical protein | DW986363 | 4 e-11 | Novel (Intracellular) |
| Mantle library clone 6D12 | Novel H. asinina mantle EST 76 | DW986364 | x | Novel (Leader sequence detected) |
| Mantle library clone 6D3 | collagen pro alpha chain | DW986365 | 4 e-26 | Intracellular with a GenBank homologue |
| Mantle library clone 6D5 | Novel H. asinina mantle EST 84 | DW986366 | x | Novel (Intracellular) |
| Mantle library clone 6D7 | 16S ribosomal RNA | DW986367 | 1 e-179 | Intracellular with a GenBank homologue |
| Mantle library clone 6D8 | Novel H. asinina mantle EST 90 | DW986368 | x | Novel (Intracellular) |
| Mantle library clone 6D9 | Novel H. asinina mantle EST 93 | DW986369 | x | Novel (Intracellular) |
| Mantle library clone 6E1 | Novel H. asinina mantle EST 95 | DW986370 | x | Novel (Intracellular) |
| Mantle library clone 6E10**(1B9) | calmodulin -1 | DW986371 | 4 e-21 | Trafficking |
| Mantle library clone 6E11 | serine/threonine protein phosphatase | DW986372 | 1 e-127 | Intracellular with a GenBank homologue |
| Mantle library clone 6E2 | Novel H. asinina mantle EST 101 | DW986373 | x | Novel (Intracellular) |
| Mantle library clone 6E4**(1G6) | Novel H. asinina mantle EST 106 | DW986374 | x | Novel (Leader sequence detected) |
| Mantle library clone 6E5 | Novel H. asinina mantle EST 110 | DW986375 | x | Novel (Leader sequence detected) |
| Mantle library clone 6E7 | Novel H. asinina mantle EST 114 | DW986376 | x | Novel (Intracellular) |
| Mantle library clone 6E8 | Novel H. asinina mantle EST 116 | DW986377 | x | Novel (Leader sequence detected) |
| Mantle library clone 6F1 | Novel H. asinina mantle EST 118 | DW986378 | x | Novel (Leader sequence detected) |
| Mantle library clone 6F11 | hypothetical protein | DW986379 | 3 e-21 | Novel (Intracellular) |
| Mantle library clone 6F12 | ribosomal protein S8 | DW986380 | 2 e-95 | Intracellular with a GenBank homologue |
| Mantle library clone 6F2 | ribosomal protein S5 | DW986381 | 1 e-119 | Intracellular with a GenBank homologue |
| Mantle library clone 6F3**(1B11) | Novel H. asinina mantle EST 127 | DW986382 | x | Novel (Leader sequence detected) |
| Mantle library clone 6F4 | Novel H. asinina mantle EST 133 | DW986383 | x | Novel (Intracellular) |
| Mantle library clone 6F5 | Novel H. asinina mantle EST 137 | DW986384 | x | Novel (Intracellular) |
| Mantle library clone 6F8 | Novel H. asinina mantle EST 142 | DW986385 | x | Novel (Intracellular) |
| Mantle library clone 6F9 | Novel H. asinina mantle EST 144 | DW986386 | x | Novel (Leader sequence detected) |
| Mantle library clone 6G1 | Novel H. asinina mantle EST 147 | DW986387 | x | Novel (Leader sequence detected) |
| Mantle library clone 6G10 | Novel H. asinina mantle EST 150 | DW986388 | x | Novel (Leader sequence detected) |
| Mantle library clone 6G11 | Novel H. asinina mantle EST 151 | DW986389 | x | Novel (Intracellular) |
| Mantle library clone 6G12 | ribulose-5-phosphate-3-epimerase | DW986390 | 6 e-87 | Intracellular with a GenBank homologue |
| Mantle library clone 6G2 | Novel H. asinina mantle EST 155 | DW986391 | x | Novel (Intracellular) |
| Mantle library clone 6G4 | FERM domain containing | DW986392 | 1 e-67 | Intracellular with a GenBank homologue |
| Mantle library clone 6G9 | lysin-like | DW986393 | 2 e -9 | Intracellular with a GenBank homologue |
| Mantle library clone 6H1 | ribosomal protein P2 | DW986394 | 4 e-21 | Intracellular with a GenBank homologue |
| Mantle library clone 6H10 | Novel H. asinina mantle EST 167 | DW986395 | x | Novel (Intracellular) |
| Mantle library clone 6H11 | ribosomal protein L15 | DW986396 | 4 e-97 | Intracellular with a GenBank homologue |
| Mantle library clone 6H12 | Novel H. asinina mantle EST 171 | DW986397 | x | Novel (Leader sequence detected) |
| Mantle library clone 6H3 | Novel H. asinina mantle EST 175 | DW986398 | x | Novel (Intracellular) |
| Mantle library clone 6H5 | Novel H. asinina mantle EST 177 | DW986399 | x | Novel (Intracellular) |
| Mantle library clone 6H8 | Novel H. asinina mantle EST 181 | DW986400 | x | Novel (Intracellular) |
| Mantle library clone 6H9 | Novel H. asinina mantle EST 184 | DW986401 | x | Novel (Intracellular) |
| Mantle library clone 7A1 | guanine nucleotide binding protein | DW986402 | 1 e-162 | Intracellular with a GenBank homologue |
| Mantle library clone 7A10 | Novel H. asinina mantle EST 6 | DW986403 | x | Novel (Leader sequence detected) |
| Mantle library clone 7A11 | Novel H. asinina mantle EST 10 | DW986404 | x | Novel (Leader sequence detected) |
| Mantle library clone 7A4 | Novel H. asinina mantle EST 18 | DW986405 | x | Novel (Intracellular) |
| Mantle library clone 7A7**(1C4) | ferritin -1 | DW986406 | 4 e-81 | Trafficking |
| Mantle library clone 7A9 | Novel H. asinina mantle EST 24 | DW986407 | x | Novel (Intracellular) |
| Mantle library clone 7B12 | Novel H. asinina mantle EST 29 | DW986408 | x | Novel (Intracellular) |
| Mantle library clone 7B3 | elongation factor 1 | DW986409 | 2 e-70 | Intracellular with a GenBank homologue |
| Mantle library clone 7B4 | Novel H. asinina mantle EST 36 | DW986410 | x | Novel (Intracellular) |
| Mantle library clone 7B6 | peroxiredoxin V | DW986411 | 3 e-61 | Intracellular with a GenBank homologue |
| Mantle library clone 7B8 | Novel H. asinina mantle EST 43 | DW986412 | x | Novel (Intracellular) |
| Mantle library clone 7C11 | Novel H. asinina mantle EST 47 | DW986413 | x | Novel (Intracellular) |
| Mantle library clone 7C12 | Laminin receptor | DW986414 | 1 e-114 | Intracellular with a GenBank homologue |
| Mantle library clone 7C3 | Novel H. asinina mantle EST 53 | DW986415 | x | Novel (Intracellular) |
| Mantle library clone 7C5 | Novel H. asinina mantle EST 60 | DW986416 | x | Novel (Intracellular) |
| Mantle library clone 7C7 | Novel H. asinina mantle EST 65 | DW986417 | x | Novel (Intracellular) |
| Mantle library clone 7C9 | ribosomal protein L23a | DW986418 | 1 e-67 | Intracellular with a GenBank homologue |
| Mantle library clone 7D1 | granulin | DW986419 | 2 e-10 | Leader sequence detected with GenBank homologue |
| Mantle library clone 7D10 | Novel H. asinina mantle EST 73 | DW986420 | x | Novel (Intracellular) |
| Mantle library clone 7D11 | Novel H. asinina mantle EST 75 | DW986421 | x | Novel (Leader sequence detected) |
| Mantle library clone 7D12 | Novel H. asinina mantle EST 77 | DW986422 | x | Novel (Intracellular) |
| Mantle library clone 7D6 | similar to fuse-binding protein-interacting repressor | DW986423 | 2 e -86 | Intracellular with a GenBank homologue |
| Mantle library clone 7D7 | Novel H. asinina mantle EST 87 | DW986424 | x | Novel (Intracellular) |
| Mantle library clone 7D8 | hypothetical protein | DW986425 | 5 e-7 | Novel (Intracellular) |
| Mantle library clone 7D9 | Novel H. asinina mantle EST 94 | DW986426 | x | Novel (Intracellular) |
| Mantle library clone 7E1 | superoxide dismutase | DW986427 | 9 e-58 | Intracellular with a GenBank homologue |
| Mantle library clone 7E10 | ribosomal protein L34 | DW986428 | 8 e-50 | Intracellular with a GenBank homologue |
| Mantle library clone 7E12 | Novel H. asinina mantle EST 99 | DW986429 | x | Novel (Intracellular) |
| Mantle library clone 7E4 | Novel H. asinina mantle EST 107 | DW986430 | x | Novel (Intracellular) |
| Mantle library clone 7E7 | Novel H. asinina mantle EST 115 | DW986431 | x | Novel (Leader sequence detected) |
| Mantle library clone 7E9 | calcium-activated potassium channel | DW986432 | 1 e-147 | Intracellular with a GenBank homologue |
| Mantle library clone 7F1* | Similar to cubilin | DW986433 | 6 e -15 | Trafficking |
| Mantle library clone 7F10 | Novel H. asinina mantle EST 119 | DW986434 | x | Novel (Intracellular) |
| Mantle library clone 7F11 | Novel H. asinina mantle EST 121 | DW986435 | x | Novel (Leader sequence detected) |
| Mantle library clone 7F12 | Novel H. asinina mantle EST 123 | DW986436 | x | Novel (Intracellular) |
| Mantle library clone 7F3 | transcription elongation factor | DW986437 | 8 e-66 | Intracellular with a GenBank homologue |
| Mantle library clone 7F4 | Novel H. asinina mantle EST 134 | DW986438 | x | Novel (Intracellular) |
| Mantle library clone 7F5 | Temptin | DW986439 | 5 e-13 | Leader sequence detected with GenBank homologue |
| Mantle library clone 7F6 | hypothetical protein | DW986440 | 5 e-15 | Novel (Leader sequence detected) |
| Mantle library clone 7F8 | hypothetical protein | DW986441 | 6 e-6 | Novel (Intracellular) |
| Mantle library clone 7F9 | Novel H. asinina mantle EST 145 | DW986442 | x | Novel (Intracellular) |
| Mantle library clone 7G1 | Novel H. asinina mantle EST 148 | DW986443 | x | Novel (Leader sequence detected) |
| Mantle library clone 7G11 | cysteine-rich protein | DW986444 | 4 e-8 | Trafficking |
| Mantle library clone 7G12 | Novel H. asinina mantle EST 153 | DW986445 | x | Novel (Intracellular) |
| Mantle library clone 7G2 | hypothetical protein | DW986446 | 4 e-8 | Novel (Intracellular) |
| Mantle library clone 7G6 | calreticulin | DW986447 | 1 e-133 | Leader sequence detected with GenBank homologue |
| Mantle library clone 7G7 | Novel H. asinina mantle EST 161 | DW986448 | x | Novel (Leader sequence detected) |
| Mantle library clone 7G9 | CCAAT/enhancer binding protein -2 | DW986449 | 4 e-8 | Transcription factor |
| Mantle library clone 7H10 | cytochrome c | DW986450 | 4 e-32 | Intracellular with a GenBank homologue |
| Mantle library clone 7H12 | Novel H. asinina mantle EST 172 | DW986451 | x | Novel (Intracellular) |
| Mantle library clone 7H2 | Novel H. asinina mantle EST 173 | DW986452 | x | Novel (Intracellular) |
| Mantle library clone 7H3 | ubiquitin | DW986453 | 3 e-77 | Intracellular with a GenBank homologue |
| Mantle library clone 7H7 | similar to cat eye syndrome | DW986454 | 7 e-51 | Novel (Intracellular) |
| Mantle library clone 7H8 | Novel H. asinina mantle EST 182 | DW986455 | x | Novel (Intracellular) |
| Mantle library clone 7H9 | Novel H. asinina mantle EST 185 | DW986456 | x | Novel (Intracellular) |
| Mantle library clone 8A1 | hypothetical protein | DW986457 | 6 e-7 | Novel (Leader sequence detected) |
| Mantle library clone 8A11 | Novel H. asinina mantle EST 11 | DW986458 | x | Novel (Intracellular) |
| Mantle library clone 8A12 | Microneme protein 4 | DW986459 | 1 e-25 | No leader sequence but extracellular |
| Mantle library clone 8A2 | Novel H. asinina mantle EST 12 | DW986460 | x | Novel (Intracellular) |
| Mantle library clone 8A3 | S-acyl fatty acid synthase thioesterase | DW986461 | 1 e-40 | Intracellular with a GenBank homologue |
| Mantle library clone 8A6 | 18S ribosomal RNA | DW986462 | 0.0 | Intracellular with a GenBank homologue |
| Mantle library clone 8B1 | Novel H. asinina mantle EST 25 | DW986463 | x | Novel (Intracellular) |
| Mantle library clone 8B11 | Novel H. asinina mantle EST 28 | DW986464 | x | Novel (Intracellular) |
| Mantle library clone 8B12 | Myosin XVIIIa | DW986465 | 1 e-55 | Trafficking |
| Mantle library clone 8B3 | Novel H. asinina mantle EST 35 | DW986466 | x | Novel (Leader sequence detected) |
| Mantle library clone 8B5 | ferritin -2 | DW986467 | 8 e-70 | Trafficking |
| Mantle library clone 8B7 | Novel H. asinina mantle EST 39 | DW986468 | x | Novel (Leader sequence detected) |
| Mantle library clone 8C10 | Notch ligand X-Delta | DW986469 | 3 e-7 | Conserved developmental signalling (intercellular) |
| Mantle library clone 8C12 | Novel H. asinina mantle EST 49 | DW986470 | x | Novel (Intracellular) |
| Mantle library clone 8C2 | L-proline 4-hydroxylase | DW986471 | 2 e-71 | Intracellular with a GenBank homologue |
| Mantle library clone 8C4 | Novel H. asinina mantle EST 56 | DW986472 | x | Novel (Leader sequence detected) |
| Mantle library clone 8C5 | BTB domain protein | DW986473 | 3 e-37 | Intracellular with a GenBank homologue |
| Mantle library clone 8C6 | 26S protease regulatory subunit 7 | DW986474 | 1 e-34 | Intracellular with a GenBank homologue |
| Mantle library clone 8C8 | Novel H. asinina mantle EST 67 | DW986475 | x | Novel (Intracellular) |
| Mantle library clone 8C9 | ribosomal protein L26 | DW986476 | 2 e-50 | Intracellular with a GenBank homologue |
| Mantle library clone 8D1 | Thiopurine S-methyltransferase | DW986477 | 4 e-25 | Intracellular with a GenBank homologue |
| Mantle library clone 8D10 | ATP synthase | DW986478 | 9 e-56 | Intracellular with a GenBank homologue |
| Mantle library clone 8D11 | EGF-like-domain | DW986479 | 1 e-9 | Intracellular with a GenBank homologue |
| Mantle library clone 8D6 | Novel H. asinina mantle EST 86 | DW986480 | x | Novel (Leader sequence detected) |
| Mantle library clone 8D8 | ribosomal protein L31 | DW986481 | 2 e-62 | Intracellular with a GenBank homologue |
| Mantle library clone 8E10 | Novel H. asinina mantle EST 97 | DW986482 | x | Novel (Leader sequence detected) |
| Mantle library clone 8E11 | Novel H. asinina mantle EST 98 | DW986483 | x | Novel (Leader sequence detected) |
| Mantle library clone 8E2 | Novel H. asinina mantle EST 102 | DW986484 | x | Novel (Intracellular) |
| Mantle library clone 8E6 | Novel H. asinina mantle EST 113 | DW986485 | x | Novel (Leader sequence detected) |
| Mantle library clone 8E8 | elongation factor | DW986486 | e-146 | Intracellular with a GenBank homologue |
| Mantle library clone 8E9 | SR protein | DW986487 | 1 e-30 | Intracellular with a GenBank homologue |
| Mantle library clone 8F10 | Novel H. asinina mantle EST 120 | DW986488 | x | Novel (Intracellular) |
| Mantle library clone 8F11 | Novel H. asinina mantle EST 122 | DW986489 | x | Novel (Leader sequence detected) |
| Mantle library clone 8F12 | hypothetical protein | DW986490 | 8 e-8 | Novel (Intracellular) |
| Mantle library clone 8F2 | Novel H. asinina mantle EST 125 | DW986491 | x | Novel (Intracellular) |
| Mantle library clone 8F3 | Novel H. asinina mantle EST 128 | DW986492 | x | Novel (Intracellular) |
| Mantle library clone 8F4 | Cell division protein kinase 7 | DW986493 | 1 e-131 | Intracellular with a GenBank homologue |
| Mantle library clone 8F5 | Novel H. asinina mantle EST 138 | DW986494 | x | Novel (Intracellular) |
| Mantle library clone 8F6 | Tigger transposable element | DW986495 | 9 e-30 | Intracellular with a GenBank homologue |
| Mantle library clone 8F7 | Glutathione peroxidase | DW986496 | 1 e-39 | Leader sequence detected with GenBank homologue |
| Mantle library clone 8F8 | hypothetical protein | DW986497 | 4 e-34 | Novel (Intracellular) |
| Mantle library clone 8F9 | Novel H. asinina mantle EST 146 | DW986498 | x | Novel (Intracellular) |
| Mantle library clone 8G1 | Eukaryotic translation initiation factor | DW986499 | 4 e-98 | Intracellular with a GenBank homologue |
| Mantle library clone 8G12 | Novel H. asinina mantle EST 154 | DW986500 | x | Novel (Intracellular) |
| Mantle library clone 8G5 | Novel H. asinina mantle EST 159 | DW986501 | x | Novel (Intracellular) |
| Mantle library clone 8G8 | Novel H. asinina mantle EST 163 | DW986502 | x | Novel (Intracellular) |
| Mantle library clone 8H1 | Novel H. asinina mantle EST 164 | DW986503 | x | Novel (Intracellular) |
| Mantle library clone 8H10 | Novel H. asinina mantle EST 168 | DW986504 | x | Novel (Leader sequence detected) |
| Mantle library clone 8H11 | Novel H. asinina mantle EST 170 | DW986505 | x | Novel (Intracellular) |
| Mantle library clone 8H2 | Actin | DW986506 | 0.0 | Intracellular with a GenBank homologue |
| Mantle library clone 8H3 | Novel H. asinina mantle EST 176 | DW986507 | x | Novel (Intracellular) |
| Mantle library clone 8H4 | hypothetical protein | DW986508 | 2 e-48 | Novel (Intracellular) |
| Mantle library clone 8H5 | Novel H. asinina mantle EST 178 | DW986509 | x | Novel (Intracellular) |
| Mantle library clone 8H8 | Novel H. asinina mantle EST 183 | DW986510 | x | Novel (Intracellular) |
| Mantle library clone 8H9 | Cytochrome C | DW986511 | 4 e-57 | Intracellular with a GenBank homologue |
| Previously isolated* | Has-Lustrin | DQ298402 | 3 e -27 | Leader sequence detected with GenBank homologue |
| Previously isolated* | Has-vm2 | DQ298397 | 8 e -15 | Leader sequence detected with GenBank homologue |

** in situ* data provided

** *in situ* data provided (riboprobe constructed from clone ID in brackets).
