# Supplementary material for: A rapidly evolving secretome builds and patterns a sea shell
Source: BMC Biol. 2006 Nov 22;4:40. doi: 10.1186/1741-7007-4-40 (PMC1676022; doi:10.1186/1741-7007-4-40)
Supplement: Additional File 2 — Table 2: Genes reported to be involved in molluskan biomineralization that were searched against the Lottia scutum genome. [file 1741-7007-4-40-S2.doc]

**Additional table 2. Genes reported to be involved in molluscan biomineralization that were searched against the *Lottia scutum* genome.**

| **Gene/protein name** | **Organism** | **Accession**  **number** | **Expression**  **domain/function** | ***Lottia scutum* trace ti number** | **E value** |
| --- | --- | --- | --- | --- | --- |
| AP7 | *Haliotis rufescens* | AAK00635 | Nacre | No significant similarity | NA |
| AP24 | *Haliotis rufescens* | AAK00634 | Nacre | No significant similarity | NA |
| Asprich AAU04811.1 - Asprich AAU04815.1 | *Atrina rigida* | AAU04811.1 - AAU04815.1 | Prismatic | No significant similarity | NA |
| Bone morphogenetic protein | *Pictada fucata* | BAD16731.1 | Unknown | 829356123 | 1 e-62 |
| Calmodulin | *Pictada fucata* | AAQ20043.1 | Unknown | 829654272 | 2 e-78 |
| Calmodulin-like protein | *Pictada fucata* | AAV73912.1 | Unknown | 829654272 | 1 e-52 |
| Calprismin | *Pinna nobilis* | P83631 | Prismatic | No significant similarity | NA |
| Ferritin like protein | *Pictada fucata* | AAQ12076.1 | Unknown | 845895363 | 3 e-19 |
| Glycine rich protein/MSI30/pMSI2 | *Pictada fucata* | BAA20465.1 | Prismatic | No significant similarity | NA |
| Glycine rich shell matrix protein MSI7 | *Pictada fucata* | AAQ08227 | Nacre and Prismatic | No significant similarity | NA |
| Insoluble protein/MSI60/pMSI1 | *Pictada fucata* | BAA20466.1 | Nacre | No significant similarity | NA |
| Lustrin A | *Haliotis rufescens* | T08852 | Nacre | 850024424 | 1 e-7 |
| Mantle gene 1 | *Pictada fucata* | AAZ76255.1 | Unknown | No significant similarity | NA |
| Mantle gene 2 | *Pictada fucata* | AAZ76256.1 | Unknown | 836424766 | 2 e-10 |
| Mantle gene 3 | *Pictada fucata* | AAZ76257.1 | Unknown | No significant similarity | NA |
| Mantle gene 4 | *Pictada fucata* | AAZ76258.1 | Unknown | 829945181 | 3 e-7 |
| Mantle gene 5 | *Pictada fucata* | AAZ76259.1 | Unknown | No significant similarity | NA |
| Mantle gene 6 | *Pictada fucata* | AAZ76260.1 | Unknown | No significant similarity | NA |
| Mantle gene 7 | *Pictada fucata* | AAZ76261.1 | Unknown | No significant similarity | NA |
| Mantle gene 8 | *Pictada fucata* | AAZ76262.1 | Unknown | No significant similarity | NA |
| Mantle gene 9 | *Pictada fucata* | AAZ22318.1 | Unknown | No significant similarity | NA |
| Mantle gene 10 | *Pictada fucata* | AAZ22319.1 | Unknown | No significant similarity | NA |
| Mantle gene 11 | *Pictada fucata* | AAZ22320.1 | Unknown | No significant similarity | NA |
| Mantle gene 12 | *Pictada fucata* | AAZ22321.1 | Unknown | 848855660 | 3 e-7 |
| Mucoperlin | *Pinna nobilis* | AAK18045 | Nacre | 844583973 | 3 e-27 |
| N14 2 pro | *Pictada fucata* | BAA83734.1 | Nacre | No significant similarity | NA |
| N14 3 pro | *Pictada fucata* | BAA83735.1 | Nacre | No significant similarity | NA |
| N14 4 pro | *Pictada fucata* | BAA83736.1 | Nacre | No significant similarity | NA |
| N14 5 pro | *Pictada fucata* | BAA83737.1 | Nacre | No significant similarity | NA |
| N14 7 pro | *Pictada fucata* | BAA83739.1 | Nacre | No significant similarity | NA |
| N66 matrix protein | *Pinctada maxima* | BAA90540.1 | Nacre | No significant similarity | NA |
| Nacrein | *Pictada fucata* | BAA11940.1 | Nacre | No significant similarity | NA |
| Nacrein | *Turbo marmoratus* | BAB91157.1 | Nacre | No significant similarity | NA |
| Pearlin | *Pictada fucata* | BAA75626.1 | Nacre | No significant similarity | NA |
| Perlucin | *Haliotis laevigata* | P82596 | Nacre | 829299731 | 1 e-7 |
| Perlustrin | *Haliotis laevigata* | P82595 | Nacre | No significant similarity | NA |
| Prismalin-14 precursor | *Pictada fucata* | Q6F4C6 | Prismatic | No significant similarity | NA |
