# Supplementary material for: A rapidly evolving secretome builds and patterns a sea shell
Source: BMC Biol. 2006 Nov 22;4:40. doi: 10.1186/1741-7007-4-40 (PMC1676022; doi:10.1186/1741-7007-4-40)
Supplement: Additional File 3 — Table 3: H. asinina mantle ESTs and biomineralization genes that share significant similarity with a Lottia scutum genomic trace. [file 1741-7007-4-40-S3.doc]

**Additional** **Table 3. *H. asinina* mantle ESTs and biomineralization genes that share significant similarity with a *Lottia scutum* genomic trace.**

| **Clone ID/protein name** | **Accession number** | ***H. asinina e*xpression**  **domain**= | ***Lottia scutum* trace**  **ti number** | **E value** |
| --- | --- | --- | --- | --- |
| *H. asinina* mantle library clone 5F3* | DW986318 | - | 829169458 | 9 e-13 |
| *H. asinina* mantle library clone 1D4* | DW986211 | - | 848775004 | 6 e-9 |
| *H. asinina* mantle library clone 1B2* | DW986194 | INF + ACOF | 836551044 | 5 e-15 |
| *H. asinina* mantle library clone 7A11* | DW986404 | - | 845973441 | 7 e-8 |
| *H. asinina* mantle library clone 6D12* | DW986364 | - | 829527461 | 4 e-19 |
| *H. asinina* mantle library clone 1E3* | DW986216 | - | 828368663 | 8 e-8 |
| *H. asinina* mantle library clone 1F3* | DW986224 | - | 828527933 | 1 e-23 |
| *H. asinina* mantle library clone 5G5* | DW986329 | - | 828306811 | 2 e-22 |
| *H. asinina* mantle library clone 6G10* | DW986388 | - | 826855967 | 4 e-31 |
| *H. asinina* mantle library clone 8D6* | DW986480 | - | 828727839 | 4 e-10 |
| *H. asinina* mantle library clone 8E10* | DW986482 | - | 839292792 | 1 e-8 |
| *H. asinina* mantle library clone 8H10* | DW986504 | - | 836615632 | 2 e-16 |
| *H. asinina* mantle library clone 3F5* | DW986260 | - | 828163540 | 8 e-7 |
| *H. asinina* mantle library clone 1H7 (salivary peroxidase) | DW986234 | INF | 829858647 | 1 e-8 |
| *H. asinina* mantle library clone 1D7 (secreted antigen) | DW986214 | INF + ACOF | 828552332 | 6 e-7 |
| *H. asinina* mantle library clone 1B9 (calmodulin) | DW986371 | INF + ACOF | 826851609 | 8 e-44 |
| *H. asinina* mantle library clone 1E4 (calcium binding protein) | DW986217 | INF + ACOF | 827242250 | 6 e-7 |
| *H. asinina* mantle library clone 7A7 (ferritin) | DW986406 | ACOF | 845895363 | 2 e-34 |
| *H. asinina* mantle library clone 5F11 (acetylserotonin O-methyltransferase) | DW986317 | ACOF | 830272451 | 4 e-23 |
| *H. asinina* mantle library clone 5B2 (six-2 homeobox) | DW986292 | ACOF | 830050251 | 9 e-46 |
| *H. asinina* mantle library clone 5D1 (mucin-like periotrophin) | DW986303 | ACOF | 828291705 | 5 e-9 |
| *H. asinina* mantle library clone 7F1 (cubilin-like) | DW986433 | ACOF | 828864999 | 3 e-7 |
| *Has-vm2* | DQ298397 | ACOF | 830418154 | 1 e-18 |
| *Has-lustrin* | DQ298402 | PZOF (Nacre) | 828700517 | 9 e-15 |
| *H. asinina* mantle library clone 1A4 | DW986185 | PZOF (Nacre) | 829880783 | 1 e-10 |

* Possess a signal peptide and share no similarity with GenBank sequences.

= Abbreviations as follows: INF, inner fold; ACOF, anterior crease of the outer fold; AZOF, anterior zone of the outer fold; PZOF, posterior zone of the outer fold.
