# Supplementary figures and images for: A rapidly evolving secretome builds and patterns a sea shell
Source: BMC Biol. 2006 Nov 22;4:40. doi: 10.1186/1741-7007-4-40 (PMC1676022; doi:10.1186/1741-7007-4-40)

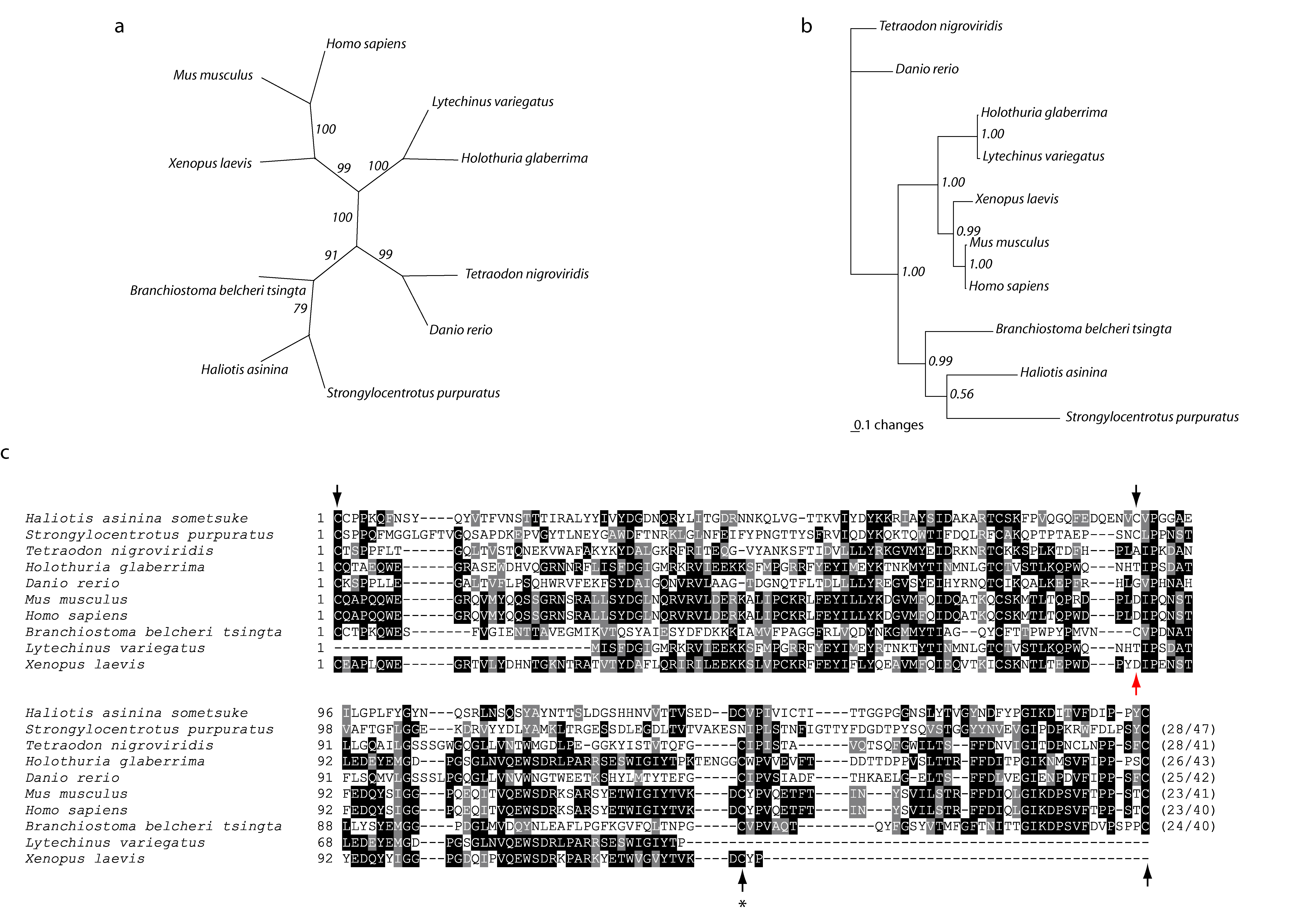

Supplement: Additional File 4 — Fig. 5: Phylogenetic analysis of Has-sometsuke (HasSom). (a) An unrooted parsimony tree (1000 bootstrap replicates) and (b) an unrooted Bayesian phylogram (200,000 generations, 1,000 burn in trees) illustrate the divergent nature of both Haliotis asinina and Strongylocentrotus purpuratus ependymin-like sequences from previously reported ependymin sequences. In agreement with a previous phylogenetic analysis [56], the echinoderms Lytechinus variegatus and Holothuria glaberima form a clade in association with the mammalian and Xenopus sequences. The amphioxus and Strongylocentrotus purpuratus sequences were not included in this previous analysis. (c) The protein alignment used to generate the trees shown in (a) and (b). Accession numbers are indicated. Alignments were created with both ClustalW and Dialign, compared and manually optimized. Arrows indicate conserved cysteine residues that are characteristic of the ependymin proteins [54]. Red arrow indicates the presence of a cysteine residue only in the H. asinina, S. purpuratus and amphioxus sequences. Asterisk indicates the loss of a cysteine residue in S. purpuratus. Figures following alignments are percentage identities and percentage positives respectively and were generated by significant pairwise bl2seq alignments between HasSom and each individual sequence. [file 1741-7007-4-40-S4.jpeg]
